# Filter Design and Performance Evaluation for Fingerprint Image Segmentation

**DOI:** 10.1371/journal.pone.0154160

**Published:** 2016-05-12

**Authors:** Duy Hoang Thai, Stephan Huckemann, Carsten Gottschlich

**Affiliations:** 1 Institute for Mathematical Stochastics, University of Goettingen, Goldschmidtstr. 7, 37077 Goettingen, Germany; 2 Statistical and Applied Mathematical Science Institute (SAMSI), 19 T. W. Alexander Drive, Research Triangle Park, 27709-4006 NC, United States of America; Bangladesh University of Engineering and Technology, BANGLADESH

## Abstract

Fingerprint recognition plays an important role in many commercial applications and is used by millions of people every day, e.g. for unlocking mobile phones. Fingerprint image segmentation is typically the first processing step of most fingerprint algorithms and it divides an image into foreground, the region of interest, and background. Two types of error can occur during this step which both have a negative impact on the recognition performance: ‘true’ foreground can be labeled as background and features like minutiae can be lost, or conversely ‘true’ background can be misclassified as foreground and spurious features can be introduced. The contribution of this paper is threefold: firstly, we propose a novel factorized directional bandpass (FDB) segmentation method for texture extraction based on the directional Hilbert transform of a Butterworth bandpass (DHBB) filter interwoven with soft-thresholding. Secondly, we provide a manually marked ground truth segmentation for 10560 images as an evaluation benchmark. Thirdly, we conduct a systematic performance comparison between the FDB method and four of the most often cited fingerprint segmentation algorithms showing that the FDB segmentation method clearly outperforms these four widely used methods. The benchmark and the implementation of the FDB method are made publicly available.

## Introduction

Nowadays, fingerprint recognition is used by millions of people in their daily life for verifying a claimed identity in commercial applications ranging from check-in at work places or libraries, access control at amusement parks or zoos to unlocking notebooks, tablets or mobile phones. Most fingerprint recognition systems are based on minutiae as features for comparing fingerprints [1]. Typical processing steps prior to minutiae extraction are fingerprint segmentation, orientation field estimation and image enhancement. The segmentation step divides an image into foreground, the region of interest (ROI), and background. Two types of error can occur in this step and both have a negative impact on the recognition rate: ‘true’ foreground can be labelled as background and features like minutiae can be lost, or ‘true’ background can be misclassified as foreground and spurious features may be introduced. It is desirable to have a method that controls both errors.

### The Factorized Directional Bandpass Method, Benchmark and Evaluation

In order to balance both errors we take the viewpoint that—loosely speaking—fingerprint images are highly determined by patterns that have frequencies only in a specific band of the Fourier spectrum (prior knowledge). Focusing on these *frequencies occuring in true fingerprint images* (FOTIs), we aim at the following goals:

1. Equally preserving all FOTIs while attenuating all non-FOTIs.
2. Removing all image artifacts in the FOTI spectrum, not due to the true fingerprint pattern.
3. Returning a (smooth) texture image containing only FOTI features from the true fingerprint pattern.
4. Morphological methods returning the ROI.

In order to meet these goals we have developed a *factorized directional bandpass* (FDB) segmentation method.

#### The FDB method

At the core of the FDB method is a classical Butterworth bandpass filter which guarantees Goal 1. Notably Goal 1 cannot fully be met by Gaussian based filtering methods such as the Gabor filter. Obviously, due to the Gaussian bell shaped curve, FOTIs would not be filtered alike. Because straightforward Fourier methods cannot cope with curvature (as could e.g. curved Gabor filters [2]) we perform separate filtering into a few isolated orientations only, via directional Hilbert transformations. The composite *directional Hilbert Butterworth bandpass filter* (DHBB) incorporates our prior knowledge about the range of possible values of ridge frequencies (between 1/3 and 1/25 pixels) or interridge distances (between 3 and 25 pixels) [2], assuming a sensor resolution of 500 DPI and that adult fingerprints are processed. In the case of adolescent fingerprints [3] or sensors with a different resolution, the images can be resized to achieve an age and sensor independent size—not only for the first segmentation step, but also for all later processing stages. Our parameters can be tuned to reach an optimal tradeoff between treating all realistic frequencies alike and avoiding Gibbs effects. Moreover we use a data friendly rectangular spectral shape of the bandpass filter employed which preserves the rectangular shape of the spatial image.

A second key ingredient is the factorization of the filter into two factors in the spectral domain, between which a thresholding operation is inserted. After preserving all FOTIs and removing all non-FOTIs in application of the first factor, all FOTI features not due to the true fingerprint pattern (which are usually less pronounced) are removed via a shrinkage operator: soft-thresholding. Note that albeit removing less pronounced FOTI features, thresholding introduces new unwanted high frequencies. These are removed, however, by application of the second factor, which also compensates for a possible phase shift due to the first factor, thus producing a smoothed image with pronounced FOTI features only.

At this stage, non-prominent FOTI features have been removed, not only outside the ROI, but also some due to true fingerprint features inside the ROI. In the final step, these “lost” regions are restored via morphological operations (convex hull after binarization and two-scale opening and closing).

**Fig 1:**
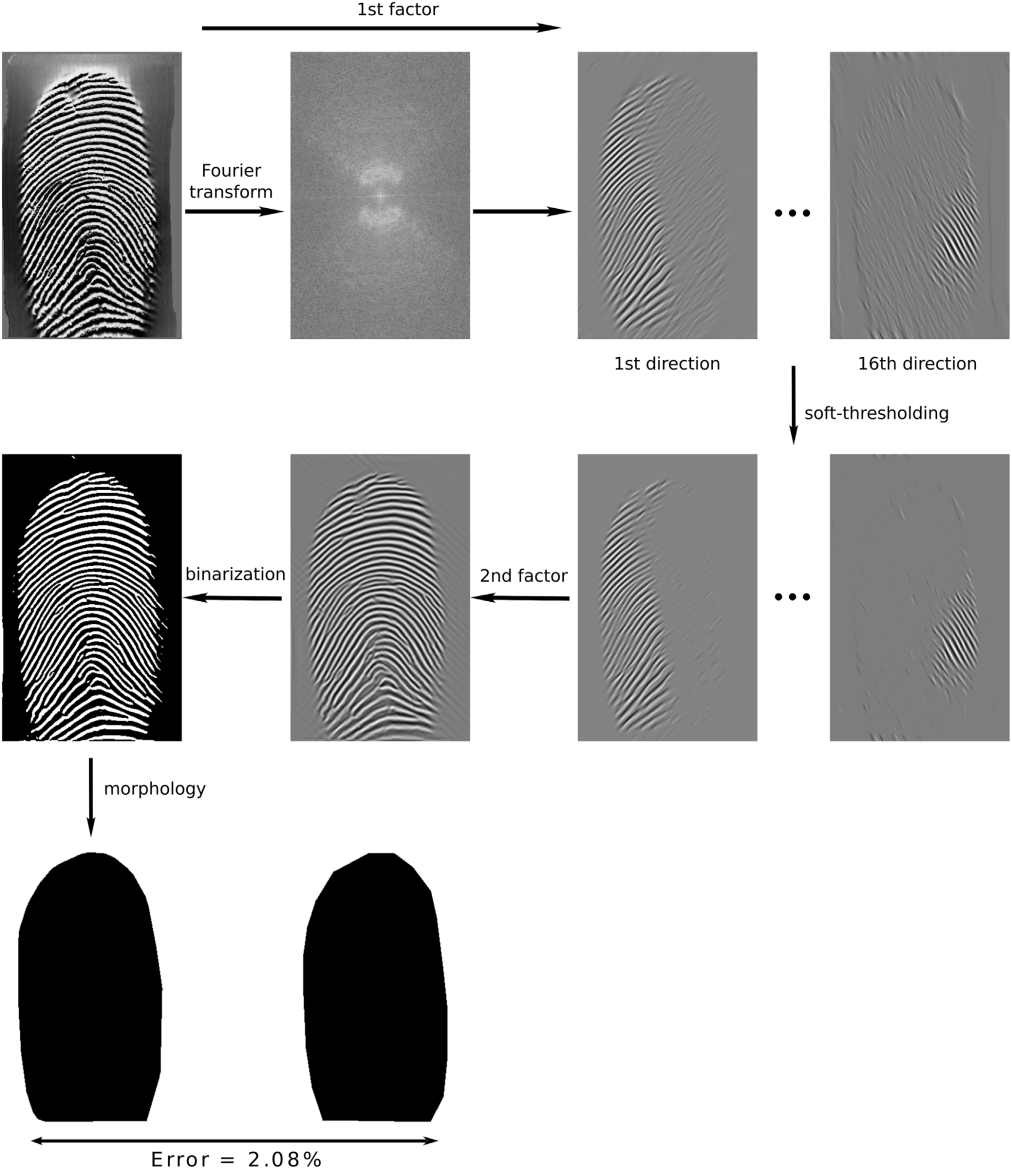
Overview over the segmentation by the FDB method: In the analysis step, the original image (top row, left) is transformed into the Fourier domain (second column) and filtered by the first DHBB factor obtaining 16 directional subbands (third and fourth columns). Next soft-thresholding is applied to remove spurious patterns (second row, third and fourth columns). In the synthesis step, the feature image (second column) is reconstructed from these subbands using the second DHBB factor. Finally, the feature image is binarized and the ROI is obtained by morphological operations. The estimated ROI (third row, left) is compared to manually marked ground truth segmentation (third row, right) in order to evaluate the segmentation performance.

The careful combination of the above ingredients in our proposed FDB method yields segmentation results far superior to existing segmentation methods. The procedure of the FDB method is illustrated in Fig 1.

#### Benchmark

In order to verify this claim, because of the lack of a suitable benchmark in the literature, we provide a manually marked ground truth segmentation for all 12 databases of FVC2000 [4], FVC2002 [5] and FVC2004 [6]. Each databases consists of 80 images for training and 800 images for testing. Overall this benchmark consists of 10560 marked segmentation images. This ground truth benchmark is made publicly available, so that other researchers can evaluate segmentation algorithms on it.

#### Evaluation against existing methods

We conduct a systematic performance comparison of widely used segmentation algorithms on this benchmark. In total, more than 100 methods for fingerprint segmentation can be found in literature. However, it remains unclear how these methods compare with each other in terms of segmentation performance and which methods can be considered as state-of-the-art. In order to remedy the current situation we chose four of the most often cited fingerprint segmentation methods and compared their performance: a method based on mean and variance of gray level intensities and the coherence of gradients as features and a neural network as a classifier [7], a method using Gabor filter bank responses [8], a Harris corner response based method [9] and an approach using local Fourier analysis [10].

### Related Work

Early methods for fingerprint segmentation include Mehtre *et al.* [11] who segment an image based on histograms of local ridge orientation and in [12] additionally the gray-level variance is considered. A method proposed by Bazen and Gerez [7] uses the local mean and variance of gray-level intensities and the coherence of gradients as features and a neural network as a classifier. Similarly Chen *et al.* [13] use block based features including the mean and variance in combination with a linear classifier. Both methods perform morphology operations for postprocessing. A method by Shen *et al.* is based on Gabor filter bank responses of blocks [8]. In [2], all pixels are regarded as foreground for which a valid ridge frequency based on curved regions can be estimated. Wu *et al.* [9] proposed a Harris corner response based method and they apply Gabor responses for postprocessing. Wang *et al.* [14] proposed to use Gaussian-Hermite moments for fingerprint segmentation. The method of Zhu *et al.* [15] uses a gradient based orientation estimation as the main feature, and a neural network detects wrongly estimated orientation and classifies the corresponding blocks as background. Chikkerur *et al.* [10] applied local Fourier analysis for fingerprint image enhancement. The method performs implicitly fingerprint segmentation, orientation field and ridge frequency estimation. Further approaches for fingerprint enhancement in the Fourier domain include Sherlock *et al.* [16], Sutthiwichaiporn and Areekul [17] and Bartůněk *et al.* [18–20]. Segmentation methods for latent fingerprints were proposed, see Zhang *et al.* [21], Nimkar and Mishra [22], Cao *et al.* [23], and the references therein. It would be of interest to see how these methods aiming at latent fingermarks perform on a benchmark of plain fingerprint images. Recently, Ferreira *et al.* [24] have proposed a method based on range filters and fuzzy C-means clustering for segmentation and binarization.

### Setup of Paper

The paper is organized as follows: in the next section, we describe the proposed method beginning with the design of the DHBB filter for texture extraction in Section Filter Design for Fingerprint Segmentation. Subsequently, the extracted and denoised texture is utilized for estimating the segmentation as described in Fingerprint Segmentation which summarizes the FDB segmentation procedure. In Section Evaluation Benchmark and Results, the manually marked ground truth benchmark is introduced and applied for evaluating the segmentation performance of four widely used algorithms and for comparing them to the proposed FDB segmentation method. The results are discussed in Section Conclusions.

## Fingerprint Segmentation by FDB Methods

Our segmentation method uses a filter transforming an input 2D image $f\left(\cdot \right)\in L_{2}\left(R^{2}\right)$ into a *feature image*

$$\tilde{f}\left(x\right)\;:=\;\sum_{l=0}^{L-1}\sum_{x\neq m\in Z^{2}}\underset{d_{l}\left[m\right]}{\underset{︸}{T\left\{{\underset{︸}{{\langle f\left(\cdot \right),\phi_{l}^{\gamma ,n}\left(\cdot -m\right)\rangle }_{L_{2}}^{pv}}}_{c_{l}\left[m\right]},\beta \right\}}}\cdot \phi_{l}^{\gamma ,n}\left(x-m\right).$$ (1)

Due to our filter design, the *L*_2_ product above as well as all convolutions, integrals and sums are understood in the principal value sense

$$\lim_{\epsilon \to 0}\int_{∥y-m∥\geq \epsilon }f\left(y\right)\cdot \phi_{l}^{\gamma ,n}\left(y-m\right)dy\;=\;{\langle f\left(\cdot \right),\phi_{l}^{\gamma ,n}\left(\cdot -m\right)\rangle }_{L_{2}}^{pv}\;=\;\left(f{*}_{pv}\phi_{l}^{\gamma ,n,\vee }\right)\left(m\right)\;.$$

Having clarified this, the symbol “pv” will be dropped in the following. At the core of Eq (1) is the DHBB filter conveyed by $\phi_{l}^{\gamma ,n}$ (*l* counts directions, *n* and *γ* are tuning parameters providing sharpness). In fact, we suitably factorize the filter conveyed by $\phi_{l}^{\gamma ,n}*\phi_{l}^{\gamma ,n,\vee }$ in the Fourier domain where $\phi_{l}^{\gamma ,n,\vee }\left(x\right):=\phi_{l}^{\gamma ,n}\left(-x\right)$ with the argument reversion operator “^∨^” and apply a thresholding procedure **T** “in the middle”. Underlying this factorization is a factorization of the bandpass filter involved. The precise filter design will be detailed in the following. Note that the directional Hilbert transform is also conveyed by a non-symmetric kernel. Reversing this transform (as well as the factor of the Butterworth) restores symmetry. It is inspired by the steerable wavelet [25–27] and to some extend similar in spirit to the curvelet transform [28], [29] and the curved Gabor filters [2]. We deal with curvature by analyzing single directions *l* separately before the final synthesis.

Via factorization, possible phase shifts are compensated and unwanted frequencies introduced by the thresholding operator are eliminated, yielding a sparse smoothed feature image. This allows for easy binarization and segmentation via subsequent morphological methods, leading to the ROI.

Note that Eq (1) can be viewed as an analog to the projection operator in sampling theory with the analysis and synthesis steps (e.g. [30]). In this vein we have the following three steps:

*Forward analysis (prediction)*: A first application of the argument reversed DHBB filter to a fingerprint image *f* corresponds to a number of directional selections in certain frequency bands of the fingerprint image giving *c*_*l*_[***m***] above.
*Proximity operator (thresholding)*: In order to remove intermediate coefficients due to spurious patterns (cf. [31]) we perform soft tresholding on the filtered grey values yielding *d*_*l*_[***m***] above.
*Backward synthesis*: Subsequently we apply the filter (non-reversed) again giving $\tilde{f}$ assembled from all subbands. A numerical comparison to other synthesis methods, summation (corresponding to a naive reconstruction) and maximal response in the appendix Comparison of the Operator in the FDB Method with the Summation and Maximum Operators, shows the superiority of this smoothing step.

Due to the discrete nature of the image $f\left[k\right]=f\left(x\right){|}_{x=k\in Z^{2}}$, we work with the discrete version of $\tilde{f}\left(x\right)$ at $x=k\in Z^{2}$ in Eq (1).

### Filter Design for Fingerprint Segmentation

The features of interest in a fingerprint image are repeated (curved) patterns which are concentrated in a particular range of frequencies after a Fourier transformation. In principle, the frequencies lower than these range’s limits correspond to homogeneous regions and those higher to small scale objects, i.e. noise, respectively. Taking this prior knowledge into account, we design an algorithm that captures these fingerprint patterns in different directional subbands in the frequency domain for extracting the texture.

In this section, we design angularpass and bandpass filters. The angularpass filter builds on iterates of the directional Hilbert transformation, a multidimensional generalization of the Hilbert transform called the Riesz transform. It can be represented via principal value convolution kernels. The bandpass filter builds on the Butterworth transform which can be represented directly via a convolution kernel. We follow here a standard technique designing a bandpass filter from a lowpass filter which has an equivalent representation in analog circuit design.

#### The *n*^th^ Order Directional Hilbert Transform of a Butterworth Bandpass

Although a fingerprint image

$$k=\left[k_{1},k_{2}\right]\mapsto f\left[k\right],\;\left\{-M,\ldots ,M\right]\times \left\{-N,\ldots ,N\right\}\to \left\{0,\ldots ,255\right\}$$

is a discrete signal observed over a discrete grid, M,N∈N we start our considerations with a signal

$$x=\left(x_{1},x_{2}\right)\mapsto f\left(x\right),\;D:=\left[-a,a\right]\times \left[-b,b\right]\to \left[0,1\right]$$

assuming values in a continuum *a*, *b* > 0. The frequency coordinates in the spectral domain will be denoted by $\omega =\left(\omega_{1},\omega_{2}\right)\in R^{2}$.

As usual, the following operators are defined first for functions *f* in the Schwartz Space of rapidly-decaying and infinitely differentiable test functions:

$$S\left(R^{d}\right)=\left\{f\in C^{\infty }\left(R^{d}\right)|\underset{x\in R^{d}}{\sup}\;\left(1+{\left|x\right|}^{m}\right)\frac{d^{n}}{dx^{n}}f\left(x\right)<+\infty \;,\;\;\forall m,n\in Z_{+}\right\},$$

and continuously extended onto

$$L_{2}\left(R^{d}\right)=\left\{f\in S\left(R^{d}\right)|{∥f∥}_{L_{2}}=\int_{R^{d}}{\left|f(x)\right|}^{2}dx<+\infty \right\}.$$

In our context we only need *d* = 1, 2. Further, we denote the Fourier and its inverse transformations by

$$\begin{array}{c} F\left[f\right]\left(\omega \right)\;=\;\int_{R^{d}}f\left(x\right)\;e^{-j\langle \omega ,x\rangle }\;dx\;=\;\hat{f}\left(\omega \right)\;,\;\;\;F^{-1}\left[f\right]\left(\omega \right)\;=\;\frac{1}{{\left(2\pi \right)}^{d}}\int_{R^{d}}\hat{f}\left(\omega \right)\;e^{j\langle \omega ,x\rangle }\;dx \end{array}$$

where *j* denotes the imaginary unit with *j*^2^ = −1.

#### Butterworth bandpass

For γ∈N and frequency bounds 0 < *ω*_*L*_ < *ω*_*H*_, setting Δ = *ω*_*H*_ − *ω*_*L*_, *p*^2^ = *ω*_*H*_ *ω*_*L*_, the one-dimensional (*d* = 1) Butterworth bandpass transform is defined via

$$\begin{array}{ccc} B[f](x) & = & F^{-1}\left[\omega \mapsto \underset{:=\hat{b}\left(\omega \right)}{\underset{︸}{\sqrt{\frac{{\left(\omega \Delta \right)}^{2\gamma }}{{\left(\omega \Delta \right)}^{2\gamma }+{\left(\omega^{2}-p^{2}\right)}^{2\gamma }}}}}\;\hat{f}\left(\omega \right)\right]\left(x\right)\;, \end{array}$$

cf. [32]. It is easy to verify that $\hat{b}\left(\omega \right)$ tends to zero for *ω* → 0 and *ω* → ∞ and has unique maximum at the geometric mean *p* with value 1. In consequence, for high values of *γ*, this filter approximates the ideal filter

$${\hat{b}}_{\text{ideal}}\left(\omega \right)=\left\{\begin{array}{cc} 1 & \;\text{if}\;\omega_{L}\leq \omega \leq \omega_{H}\\ 0 & \;\text{else}\; \end{array}\right..$$

The ideal filter, however, suffers from the Gibbs effect. Letting $t=\frac{{\left(j\omega \right)}^{2}+p^{2}}{j\omega \Delta }$, we factorize the bandpass Butterworth as

$${\hat{b}}^{2}\left(\omega \right)=\frac{1}{{\left(-1\right)}^{\gamma }{\left(t^{2}\right)}^{\gamma }+1}=\frac{1}{{\left(-1\right)}^{\gamma }\prod_{k=1}^{\gamma }\left(t^{2}-t_{k}^{2}\right)}=\underset{H(t)}{\underset{︸}{\prod_{k=1}^{\gamma }\frac{1}{t-t_{k}}}}\cdot \underset{H(-t)}{\underset{︸}{\prod_{k=1}^{\gamma }\frac{1}{-t-t_{k}}}},$$

with *t*_*k*_ = *e*^*πj*(*γ* + 2*k* − 1)/2*γ*^ (*k* = 1, …, *γ*), the negative squares of which representing the *γ* different complex roots of (−1). Then, with the below complex valued factor of $0\leq {\hat{b}}^{2}\left(\omega \right)=B\left(j\omega \right)\cdot B\left(-j\omega \right)$ called the transfer function,

$$H\left(t\right)\;=\;H\left(\frac{{\left(j\omega \right)}^{2}+p^{2}}{j\omega \Delta }\right)\;=\;\prod_{k=1}^{\gamma }\frac{\Delta (j\omega )}{{\left(j\omega \right)}^{2}-\Delta t_{k}\left(j\omega \right)+p^{2}}\;:=\;B\left(j\omega \right)\;,$$

we use the approximation: $j\omega \;=\;\log e^{j\omega }\;\approx \;2\frac{e^{j\omega }-1}{e^{j\omega }+1}$ to obtain

$$B\left(j\omega \right)\;\approx \;\prod_{k=1}^{\gamma }\frac{2\Delta (e^{2j\omega }-1)}{\left(4+p^{2}-2\Delta t_{k}\right)e^{2j\omega }+\left(2p^{2}-8\right)e^{j\omega }+\left(4+p^{2}+2\Delta t_{k}\right)}:=B^{\gamma }\left(e^{j\omega }\right).$$

**Fig 2:**
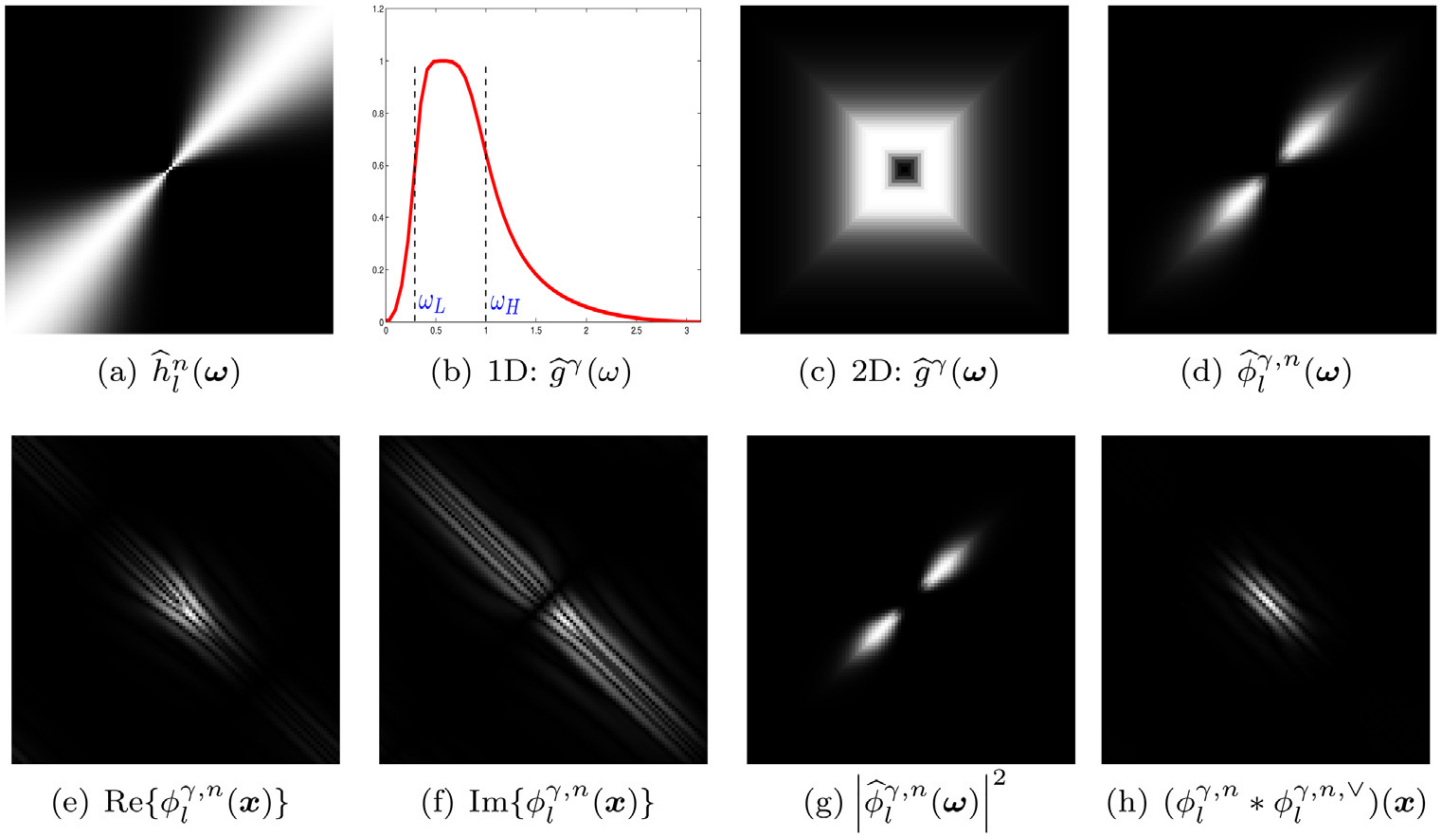
Image (a) displays a 1D Butterworth bandpass filter (blue) and its approximation (red). Image (b) shows the 2D Butterworth bandpass filter ${\hat{g}}^{\gamma }\left(\omega \right)$ at *n* = 20, *θ* = 0, *γ* = 2, and the corresponding DHBB filter in the Fourier and spatial domains (c, d) for the approximation by the bilinear transform. Image (e) visualizes the 2D version of the original filter and the corresponding DHBB filter (f, g).

This approximation is often called the bilinear transform, which turns out to reduce the frequency bandwidth of interest, cf. Fig 2.

The 1D filter *B*^*γ*^(*e*^*iω*^) is then generalized to a 2D domain. The McClellan transform [33–36], would be one favorable method. Also, recently, bandpass filtering with a radial filter in the Fourier domain has been proposed by [37], [38] and [39] et al. for enhancing fingerprint images. However, for a simpler reconstruction of 2D filter and a data-friendly alternative to the polar tiling of the frequency plane, a Cartesian array is used instead (see [28], [29], [40], [41]).

Thus, on a rectangular domain *D* = [−*a*, *a*] × [−*b*, *b*] with common cuttoff frequencies 0 < *ω*_*L*_ < *ω*_*H*_ and the two characteristic functions

$$\chi_{h}\left(\omega_{1},\omega_{2}\right):=\left\{\begin{array}{cc} 1 & \;\text{if}\;b|\omega_{1}\left|\geq a\right|\omega_{2}|\\ 0 & \;\text{else} \end{array}\right.\;,\;\chi_{v}\left(\omega_{1},\omega_{2}\right):=\left\{\begin{array}{cc} 1 & \;\text{if}\;b|\omega_{1}\left|\leq a\right|\omega_{2}|\\ 0 & \;\text{else} \end{array}\right.\;$$

(see Fig 3), define

$$\begin{array}{c} {\hat{g}}^{\gamma }\left(\omega_{1},\omega_{2}\right)=B^{\gamma }\left(e^{j\omega_{1}}\right)\chi_{h}\left(\omega_{1},\omega_{2}\right)+B^{\gamma }\left(e^{j\omega_{2}}\right)\chi_{v}\left(\omega_{1},\omega_{2}\right) \end{array}$$ (2)

as the spectrum of our two-dimensional Butterworth filter *g*^***γ***^(***x***). Note that since ${\hat{g}}^{\gamma }\left(\omega \right)\in L_{2}\left(R^{2}\right)$, there is a well defined $g^{\gamma }\left(x\right)=F^{-1}[{\hat{g}}^{\gamma }\left(\omega \right)]\left(x\right).$

**Fig 3:**
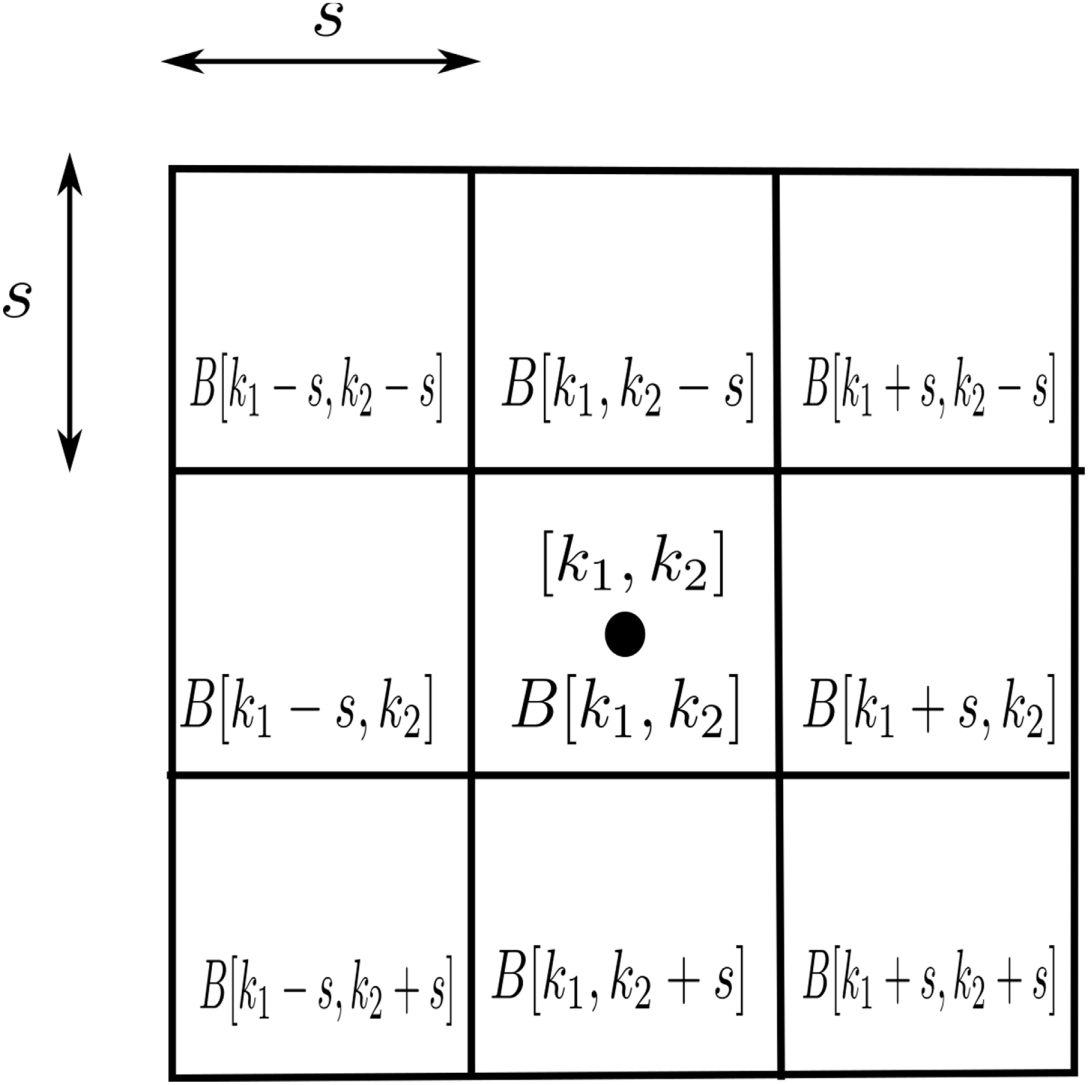
The indicator functions in the horizontal direction (a) and vertical direction (b).

**Fig 4:**
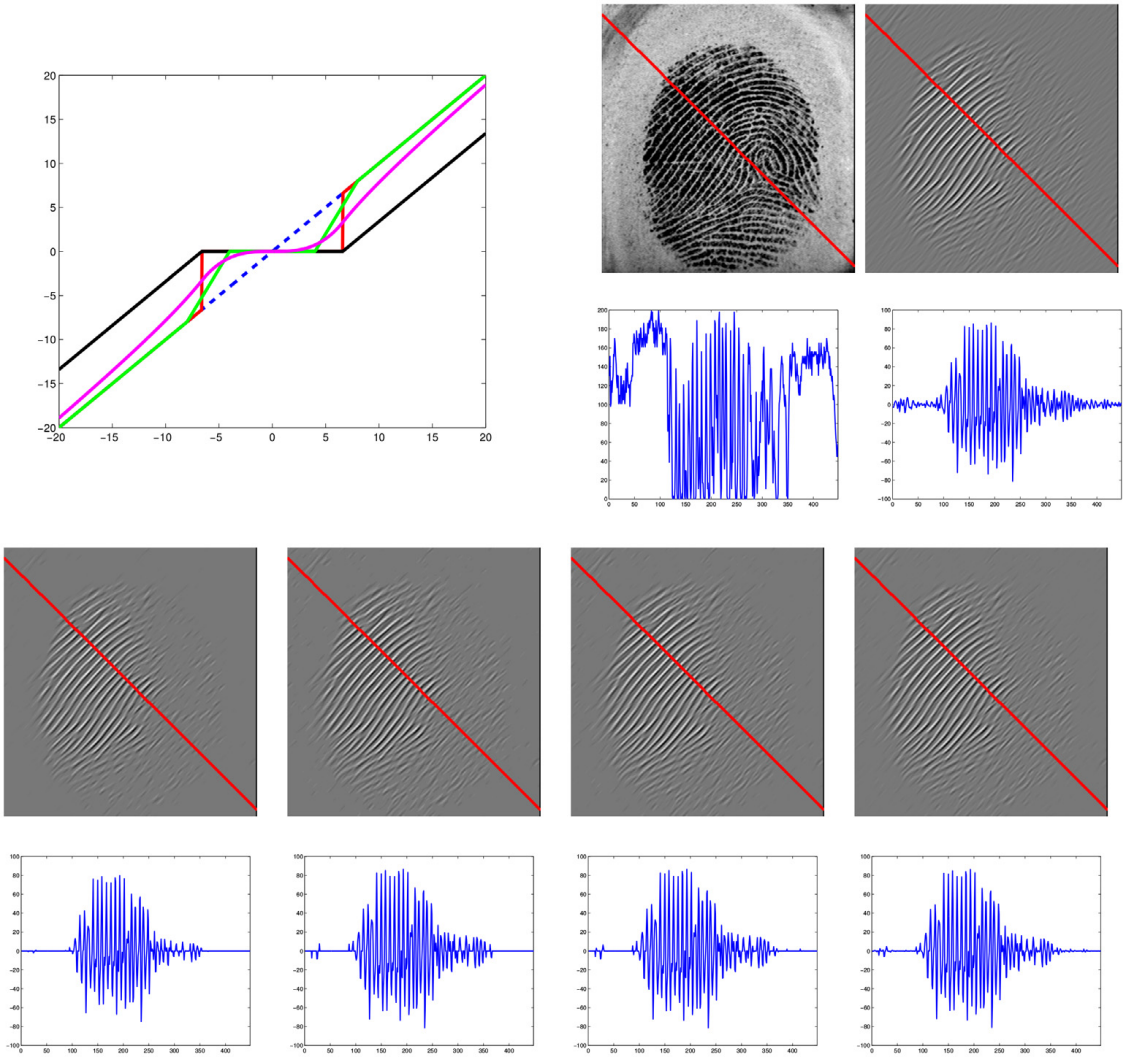
Image (a) displays the angularpass filter ${\hat{h}}_{l}^{n}\left(\omega \right)$ with *θ* = 13*π*/16, *n* = 20. Images (b-c) show the 1D and 2D Butterworth bandpass filters ${\hat{g}}^{\gamma }\left(\omega \right)$ and ${\hat{g}}^{\gamma }\left(\omega \right)$ with *ω*_*L*_ = 0.3, *ω*_*H*_ = 1, *γ* = 2, (d) the spectrum of the DHBB filter ${\hat{\phi }}_{l}^{\gamma ,n}\left(\omega \right)$. Images (e-f) visualize the real and imaginary part of the DHBB filter $\phi_{l}^{\gamma ,n}\left(x\right)$. Images (g-h) display the squared magnitude of the spectrum of the DHBB in the frequency and spatial domains which acts somewhat like a Gabor filter.

Fig 4(b) and 4(c) show an example of the 1D and 2D Butterworth bandpass filters.

#### *n*-th order directional Hilbert transformations

For more detail on the Hilbert transform H and the Riesz transform R, we refer the reader to the literature for an in-depth discussion [25], [27], [42], [43], [44], [45–47], [48], and [49].

Consider a vector $u\in R^{d}$ and set and compute, respectively, for $x\in R^{d}$

$$R\left[f\right]\left(x\right):=F^{-1}\left[\omega \mapsto \underset{:=\;\hat{h}\left(\omega \right)}{\underset{︸}{-j\;\frac{\omega }{∥\omega ∥}}}\;\hat{f}\left(\omega \right)\right]\left(x\right)$$ (3)

$$H_{u}\left[f\right]\left(x\right):=\langle u,R\left[f\right]\left(x\right)\rangle \;=\;F^{-1}\left[\omega \mapsto \underset{:=\;{\hat{h}}_{u}\left(\omega \right)}{\underset{︸}{-j\;\frac{\langle u,\omega \rangle }{∥\omega ∥}}}\;\hat{f}\left(\omega \right)\right]\left(x\right)$$

$$\underset{n-\text{times}}{\underset{︸}{H_{u}\ldots H_{u}}}\left[f\right]\left(x\right)\;=\;H_{u}^{n}\left[f\right]\left(x\right)=F^{-1}\left[\omega \mapsto \underset{{\hat{h}}_{u}^{n}\left(\omega \right)}{\underset{︸}{{\left(-j\right)}^{n}\;\frac{{\langle u,\omega \rangle }^{n}}{{∥\omega ∥}^{n}}}}\;\hat{f}\left(\omega \right)\right]\left(x\right),\;n\in N$$ (4)

The first line Eq (3) called the Riesz transform has a representation as a principal value integral

$$\begin{array}{ccc} R[f](x) & = & \left(f{*}_{pv}h\right)\left(x\right)\;=\;\lim_{\epsilon \to 0}\int_{∥y-x∥\geq \epsilon }f\left(x-y\right)\;h\left(y\right)\;dy \end{array}$$

where

$$h\left(y\right):=\left\{\begin{array}{cc} \frac{y}{{∥y∥}^{d+1}}\frac{\Gamma \left(\frac{d+1}{2}\right)}{\pi^{\frac{d+1}{2}}} & \;\text{for}\;d>1\\ \frac{1}{\pi y} & \;\text{for}\;d=1 \end{array}\right.$$

Setting *h*_***u***_(***y***) = 〈***u***, *h*(***y***)〉 and $h_{u}^{n}\left(y\right)=(h_{u}{*}_{pv}\ldots {*}_{pv}h_{u})\left(y\right)$ we haven for the third line Eq (4) called the *n*-order directional Hilbert transform that

$$H_{u}^{n}\left[f\right]\left(x\right)\;=\;f{*}_{pv}h_{u}^{n}\left(x\right)\;.$$

Since $-1\leq \frac{\langle u,\omega \rangle }{∥\omega ∥}\leq 1$ and high powers preserve the values near ±1 while forcing all other values in (−1, 1) towards 0, this filter gives roughly the same result as an inverse Fourier transform of a convolution of the signal’s Fourier transform with

$$A_{\alpha ,u}\left(\omega \right)=\left\{\begin{array}{cc} 1 & \;\text{if}\;\frac{\left|\langle u,\omega \rangle \right|}{∥\omega ∥}\geq \cos\alpha \\ 0 & \;\text{else}\; \end{array}\right.$$

for small *α* > 0. The directional Hilbert transform, however, suffers less from a Gibbs effect than this sharp cutoff filter.

In 2D, the direction vector is ***u*** = [cos(*θ*), sin(*θ*)]^*T*^ with the discretized $\theta =\frac{\pi l}{L}\in \left[0,\pi \right)$ and *l* = 0, 1, …, *L* − 1, where L∈N is the total number of orientation. Rewrite the impulse response of the n-th∈N order directional Hilbert transform in Eq (4) as

$${\hat{h}}_{u}^{n}\left(\omega_{1},\omega_{2}\right)\;=\;{\left[-j\cos\left(\tan_{2}^{-1}\left(\frac{\omega_{2}}{\omega_{1}}\right)-\frac{\pi l}{L}\right)\right]}^{n}\;:=\;{\hat{h}}_{l}^{n}\left(\omega_{1},\omega_{2}\right).$$ (5)

Putting together (Eqs (2), (4) and (5)), for a fixed bandpass *ω*_*L*_ < *ω*_*H*_ and *L* directional subbands we have thus the DHBB filter of order *γ*, *n*:

$$H_{u}^{n}\left[g^{\gamma }\right]\left(x\right)\;=\;F^{-1}\left[\omega \mapsto \underset{:={\hat{\phi }}_{l}^{\gamma ,n}\left(\omega \right)}{\underset{︸}{{\hat{h}}_{l}^{n}\left(\omega \right)\cdot {\hat{g}}^{\gamma }\left(\omega \right)}}\right]\left(x\right)\;:=\;\phi_{l}^{\gamma ,n}\left(x\right).$$ (6)

#### Thresholding

For given *β* > 0, soft-thresholding is defined as follows

$$x\mapsto T\left(x,\beta \right)=\frac{x}{\left|x\right|}\cdot \max\left(|x|-\beta ,0\right).$$ (7)

**Fig 5:**
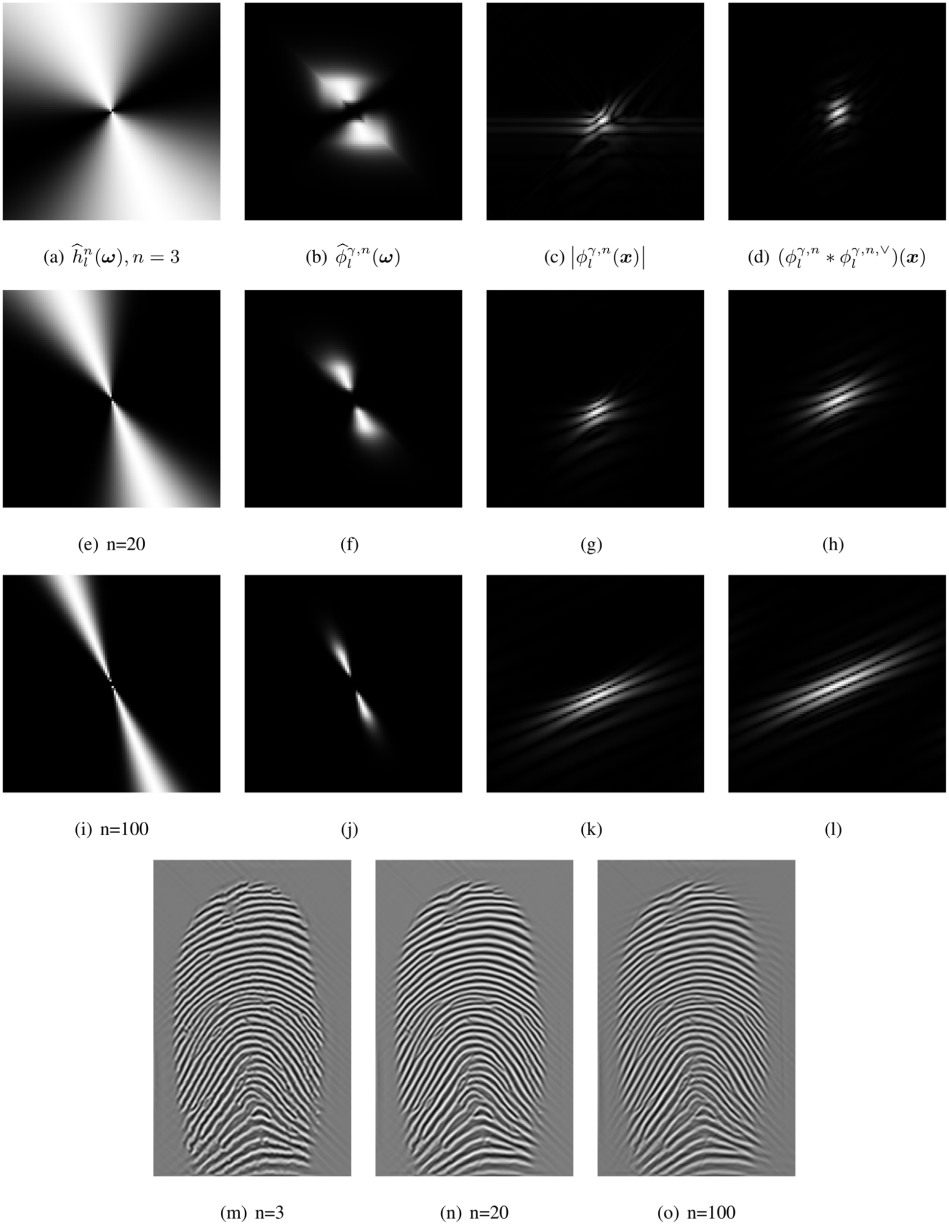
Four typical thresholding functions (red: hard, black: soft, green: semi-soft, magenta: nonlinear) are compared (top left). The following six pairs show an image and the visualization of the corresponding 1D cross section along the red line. F.l.t.r and top to bottom: the original image *f*[***k***], the coefficient *c*_*l*_[***k***] and the thresholded coefficients *d*_*l*_[***k***] for the soft, hard, semisoft and nonlinear thresholding operators. Comparing the four cross sections in the bottom row, we observe that soft-thresholding achieves the sparsest solution.

Thus, the thresholded coefficients are *d_l_* [***m***] = **T**{*c_l_* [***m***], *β*}. Note that *d*_*l*_[***m***] is a solution of the *ℓ*_1_-shrinkage minimization problem

$$\min_{u}\left\{\beta ∥u{∥}_{\ell_{1}}+\frac{1}{2}∥u-c_{l}{∥}_{\ell_{2}}^{2}\right\}\;$$

yielding soft-thresholding (cf. [50]). Fig 5 visualizes the effect of the soft-thresholding and the comparison with the others (such as: hard [50], semi-soft [51] and nonlinear [52] thresholding operators).

### Fingerprint Segmentation

After having designed the FDB filter, let us now ponder on parameter selection, image binarization and morphological processing.

#### Parameter Choice for Texture Extraction

A fingerprint image will be rescaled such that its oscillation pattern stays in a specific range in the Fourier domain, the coordinates of which are *ω*_*i*_ = [−*π*, *π*], *i* ∈ {1, 2}. For choosing the cutoff frequencies *ω*_*L*_ and *ω*_*H*_, we incorporate our prior knowledge about adult fingerprint images at resolution of 500 DPI: Valid interridge distances remain in a known range approximately from 3 to 25 pixels [2]. This corresponds exactly to *ω*_*H*_ = 1 as a limit for high frequencies. A limit of *ω*_*L*_ = 0.3 for low frequencies of the Butterworth bandpass filter corresponds to an interridge distance of about 12 pixels. The range |*ω*_*i*_| ∈ [*ω*_*H*_, *π*] contains the small scale objects which are considered as noise. The range |*ω*_*i*_| ∈ [0, *ω*_*L*_] contains the low frequency objects, corresponding to homogeneous regions.

**Fig 6:**
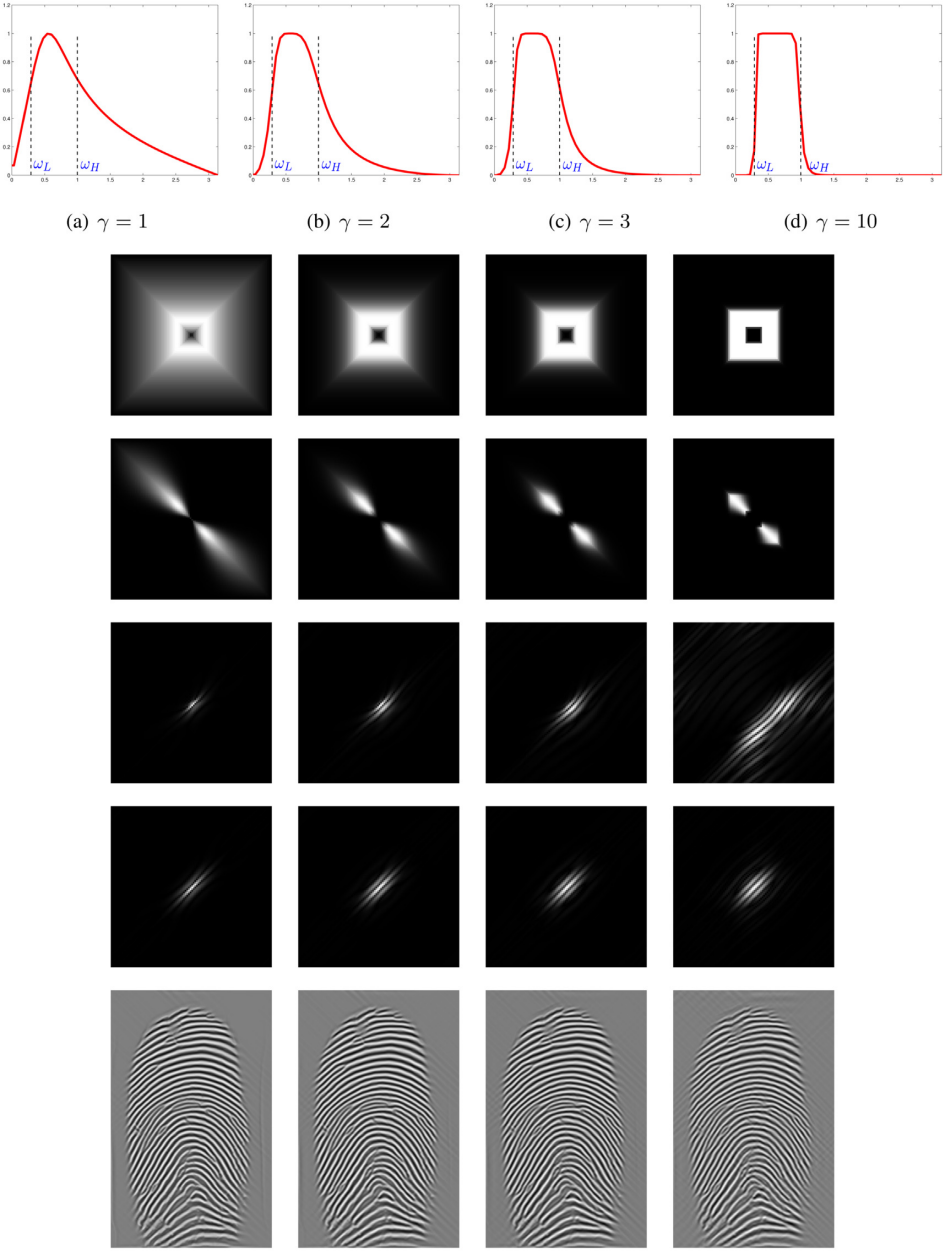
Angular bandpass ${\hat{h}}_{l}^{n}\left(\omega \right)$ at $\theta =\frac{7\pi }{16},\gamma =3$ and different orders *n* ∈ {3, 20, 100} and their responses (last row).

The number of directions *L* in and the order *n* of the directional Hilbert transform involves a tradeoff between the following effects. We observe that with increased order *n* the filter’s shape becomes thinner in the Fourier domain. Although this sparsity smooths the texture image in the spatial domain, in order to fully cover all FOTIs, *L* needs to grow with *n*. However, a disadvantage of choosing large *n* and *L* is that errors occur on the boundary due to the over-smoothing effect as illustrated in Fig 6 (o).

**Fig 7:**
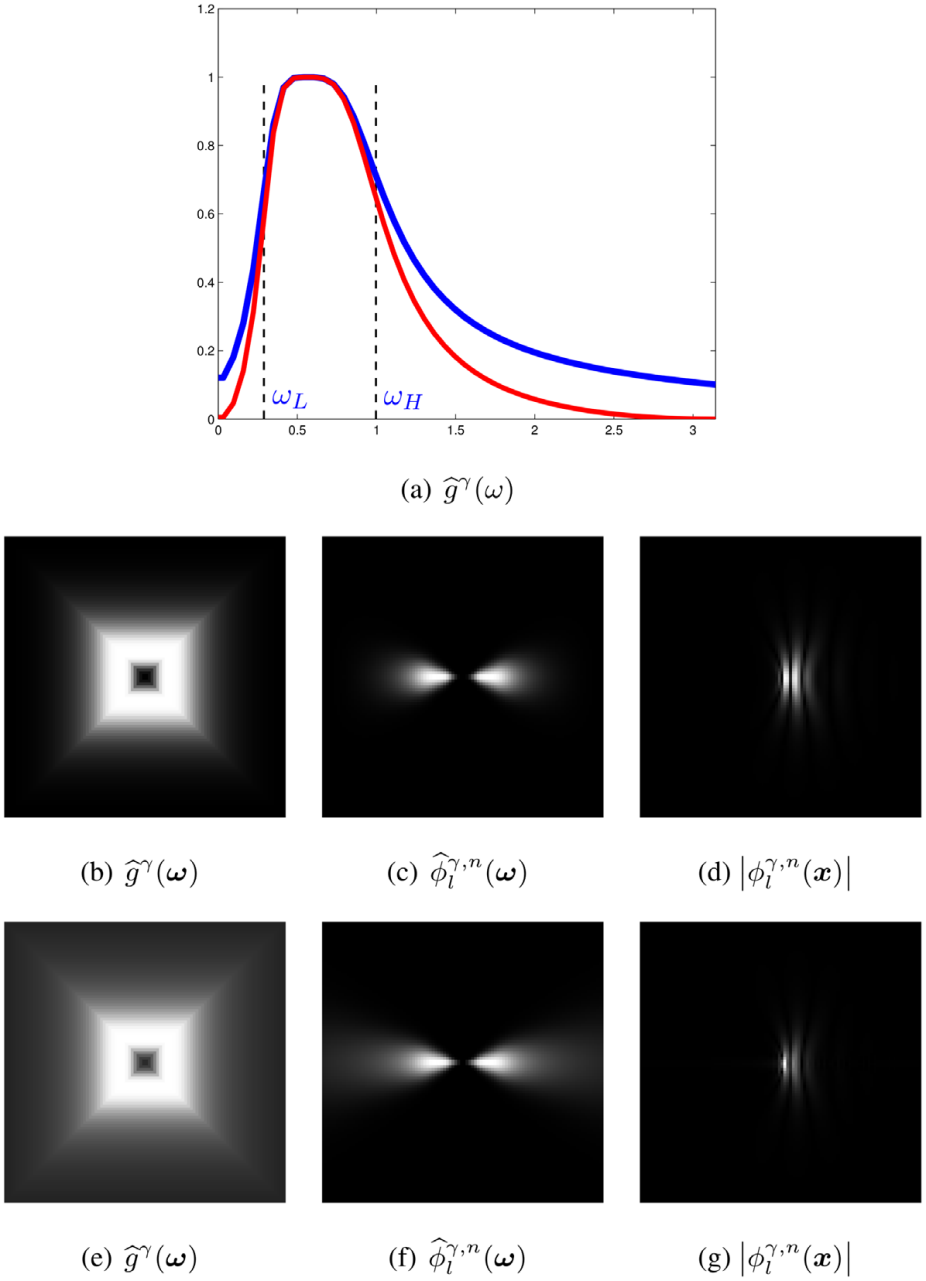
Butterworth bandpass filter ${\hat{g}}^{\gamma }\left(\omega \right)$ at *ω*_*L*_ = 0.3, *ω*_*H*_ = 1 and different *γ*, angular bandpass filter with $n=20,L=16,\theta =\frac{5\pi }{16}$, and their responses. 1^st^ row: 1D Butterworth, 2^nd^ row: 2D Butterworth, 3^rd^ row: ${\hat{\phi }}_{l}^{\gamma ,n}\left(\omega \right)$, 4^th^ row: $\left|\phi_{l}^{\gamma ,n}\left(x\right)\right|$, 5^th^ row: $\left(\phi_{l}^{\gamma ,n}*\phi_{l}^{\gamma ,n,\vee }\right)\left(x\right)$, 6^th^ row: their responses.

The next parameter to select is the order of the Butterworth filter *γ*. An illustration of the filter for different orders *γ* ∈ {1, 2, 3, 10} and with cutoff frequencies *ω*_*L*_ = 0.3 and *ω*_*H*_ = 1 is shown in Fig 7, its bilinear approximation in Fig 2. As *γ* increases the filter becomes sharper. For very large values of *γ*, it approaches the ideal filter which is known to cause the unfavorable Gibbs effect.

The thresholding value *β* separates large coefficients corresponding to the fingerprint pattern (FOTIs) (which are slightly attenuated due to soft-thresholding) from small coefficients corresponding to non-FOTIS and FOTIs which are not features due to the fingerprint pattern (these are eliminated). On the one hand, if *β* is chosen too large, more prominent parts of true fingerprint tend to be removed. On the other hand, if *β* is chosen too small, not all all unwanted features (as above) are removed which may cause segmentation errors.

In order to find good trade-offs, as described above, *n*, *L*, *γ* and *β* are trained as described in Section Experimental Results. In fact, since different fingerprint sensors have different properties, *β* is adaptively adjusted to the intensity of coefficients in all subbands as

$$\beta =C\cdot \max_{l,m}\left\{c_{l}\left[m\right]\right\}.$$ (8)

Thus, instead of *β*, *C* is trained for each sensor.

#### Texture Binarization

In the first step, the texture is decomposed by the operator Eq (1) to obtain the reconstructed image $\tilde{f}\left[k\right]$. Then, $\tilde{f}\left[k\right]$ is binarized using an adaptive threshold adjusted to the intensity of $\tilde{f}\left[k\right]$. Thus, the threshold is chosen as $C\cdot \max_{k}\left(\tilde{f}\left[k\right]\right)$, with *C* from Eq (8). If $\tilde{f}\left[k\right]$ is larger than this threshold, it will be set to 1 (foreground), otherwise, it is set to 0 (background) as illustrated in Fig 1.

$${\tilde{f}}_{\text{bin}}\left[k\right]=\left\{\begin{array}{cc} 1, & \tilde{f}\left[k\right]\geq C\cdot \max_{k}\left(\tilde{f}\left[k\right]\right)\\ 0, & \text{else} \end{array}\right.,\;\forall k\in \Omega$$

#### Morphological Processing

**Fig 8:**
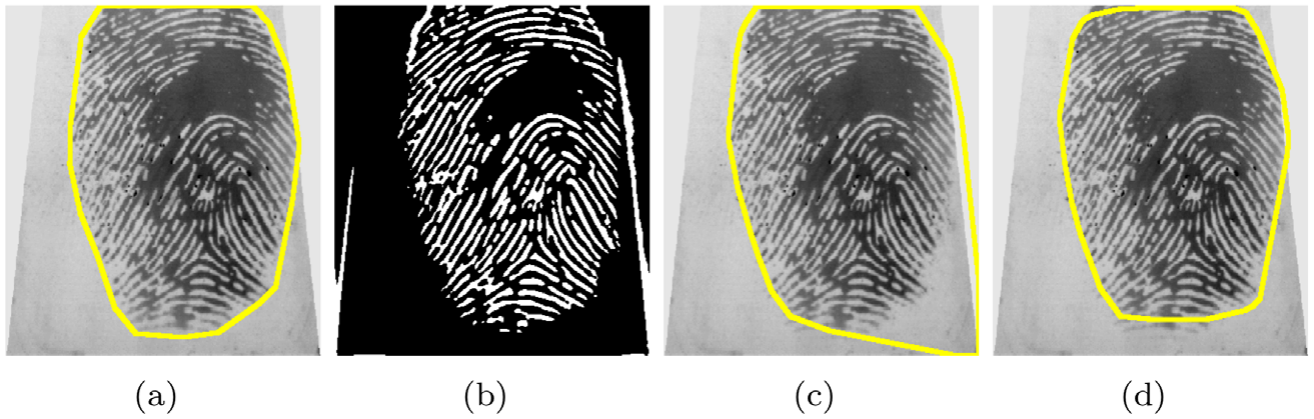
The morphological element.

In this final phase, we apply mathematical morphology (see Chapter 13 in [53]), to decide for each pixel whether it belongs to the foreground or background. Firstly, at each pixel ${\tilde{f}}_{\text{bin}}\left[k_{1},k_{2}\right]\in \left\{0,1\right\}$, we build an *s* × *s* block centered at (*k*_1_, *k*_2_) and 8 neighboring blocks (cf. Fig 8). Then, for each block, we count the white pixels and check whether their number exceeds the threshold $\frac{s^{2}}{t}$ with another parameter *t* > 0. If at least *b* blocks are above threshold, the pixel [*k*_1_, *k*_2_] is considered as foreground.

$${\tilde{f}}_{\text{dilate}}\left[k_{1},k_{2}\right]=\left\{\begin{array}{cc} 1, & \#\left\{\sum B_{\left[k_{1}+m,k_{2}+m\right]}\geq \frac{s^{2}}{t},m\in \left\{-s,0,s\right\}\right\}\geq b\\ 0, & else \end{array}\right.$$ (9)

**Fig 9:**
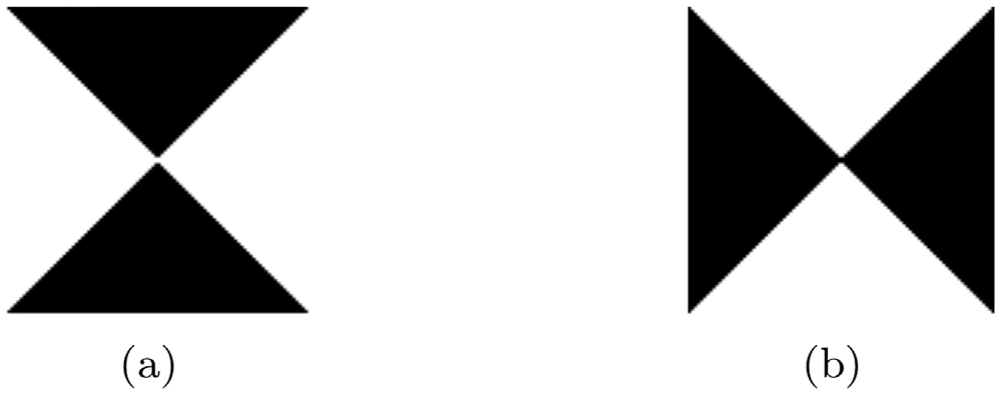
The ground truth segmentation (a) and the binarized texture image (b) for an example fingerprint. Applying a standard morphology operation like closing (dilation followed by erosion) instead of the proposed method connects in this example the white fingerprint texture with structure noise close to the margin of the texture and the result is a defective segmentation (c). The proposed morphology avoids this undesired effect by considering neighborhoods on two scales: cells of size *s* × *s* pixels and blocks of 3 × 3 cells.

Then, the largest connected white pixel component is selected by a region filling method. Its convex hull is then the ROI. For better visualization we have inverted white and black, i.e. display the background by white pixels and the ROI by black pixels, cf. Fig 1. Fig 9 illustrates the effect of the morphological operator.

## Evaluation Benchmark and Results

The databases of FVC2000, 2002 and 2004 [4–6] are publicly available and established benchmarks for measuring the verification performance of algorithms for image enhancement and fingerprint matching. Each competition comprises four databases: three of which contain real fingerprints acquired by different sensors and a database of synthetically generated images (DB 4 in each competition).

It has recently been shown that real and synthetic fingerprints can be discriminated with very high accuracy using minutiae histograms (MHs) [54]. More specifically, by computing the MH for a minutiae template and then computing the earth mover’s distance (EMD) [55] between the template MH and the mean MHs for a set of real and synthetic fingerprints. Classification is simply performed by choosing the class with the smaller EMD.

The nine databases containing real fingerprints have been obtained by nine different sensors and have different properties. The fingerprint image quality ranges from good quality images (especially FVC2002 DB1 and DB2) to low quality images which are more challenging to process (e.g. the databases of FVC2004). Some aspects of image quality concern both the segmentation step and the overall verification process, other aspects pose problems only for later stages of the fingerprint verification procedure, but have no influence on the segmentation accuracy.

Aspects of fingerprint image quality which complicate the segmentation:

- dryness or wetness of the finger
- a ghost fingerprint on the sensor surface
- small scale noise
- large scale structure noise
- image artifacts e.g. caused by reconstructing a swipe sensor image
- scars or creases interrupting the fingerprint pattern

Aspects of fingerprint image quality which make an accurate verification more difficult, but do not have any influence on the fingerprint segmentation step:

- distortion, nonlinear deformation of the finger
- small overlap area between two imprints

Each of the 12 databases contains 110 fingers with 8 impressions per finger. The training set consists of 10 fingers (80 images) and the test set contains 100 fingers (800 images). In total there are 10560 fingerprint images giving 10560 marked ground truth segmentations for training and testing.

### Experimental Results

#### Segmentation Performance Evaluation

Let *N*_1_ and *N*_2_ be the width and height of image *f*[***k***] in pixels. Let *M*_*f*_ be number of pixels which are marked as foreground by human expert and estimated as background by an algorithm (missed/misclassified foreground). Let *M*_*b*_ be number of pixels which are marked as background by human expert and estimated as foreground by an algorithm (missed/misclassified background). The average total error per image is defined as

$$Err=\frac{M_{f}+M_{b}}{N_{1}\times N_{2}}.$$ (10)

The average error over 80 training images is basis for the parameter selection. In Table 1, we report the average error over all other 800 test images for each database and for each algorithms.

**Table 1:** Error rates (average percentage of misclassified pixels averaged over 800 test images per database) computed using the manually marked ground truth segmentation and the estimated segmentation by these methods: a Gabor filter bank (GFB) response based method by Shen *et al.* [8], a Harris corner response (HCR) based approach by Wu *et al.* [9], a method by Bazen and Gerez using local gray-level mean, variance and gradient coherence (MVC) as features [7], a method applying short time Fourier transforms (STFT) by Chikkerur *et al.* [10] and the proposed method based on the factorized directional bandpass (FDB).

FVC	DB	GFB [8]	HCR [9]	MVC [7]	STFT [10]	FDB
2000	1	13.26	11.15	10.01	16.70	5.51
	2	10.27	6.25	12.31	8.88	3.55
	3	10.63	7.80	7.45	6.44	2.86
	4	5.17	3.23	9.74	7.19	2.31
2002	1	5.07	3.71	4.59	5.49	2.39
	2	7.76	5.72	4.32	6.27	2.91
	3	9.60	4.71	5.29	5.13	3.35
	4	7.67	6.85	6.12	7.70	4.49
2004	1	5.00	2.26	2.22	2.65	1.40
	2	11.18	7.54	8.06	9.89	4.90
	3	8.37	4.96	3.42	9.35	3.14
	4	5.96	5.15	4.58	5.18	2.79
Avg.		8.33	5.78	6.51	7.57	3.30

#### Parameter Selection

Experiments were carried out on all 12 databases and are reported in Table 1. For each method listed in Table 1, the required parameters were trained on each of the 12 training sets: the choice of the threshold values for the Gabor filter bank based approach by Shen *et al.* [8], and the threshold values for the Harris corner response based method by Wu *et al.* [9]. The parameters of the method by Bazen and Gerez are chosen as described in [7]: the window size of the morphology operator and the weights of the perceptron which are trained in 10^4^ iterations due to the large number of pixels in the training database. For the method of Chikkerur *et al.*, we used the energy image computed by the implementation of Chikkerur, performed Otsu thresholding and mathematical morphology as explained in [53].

**Fig 10:**
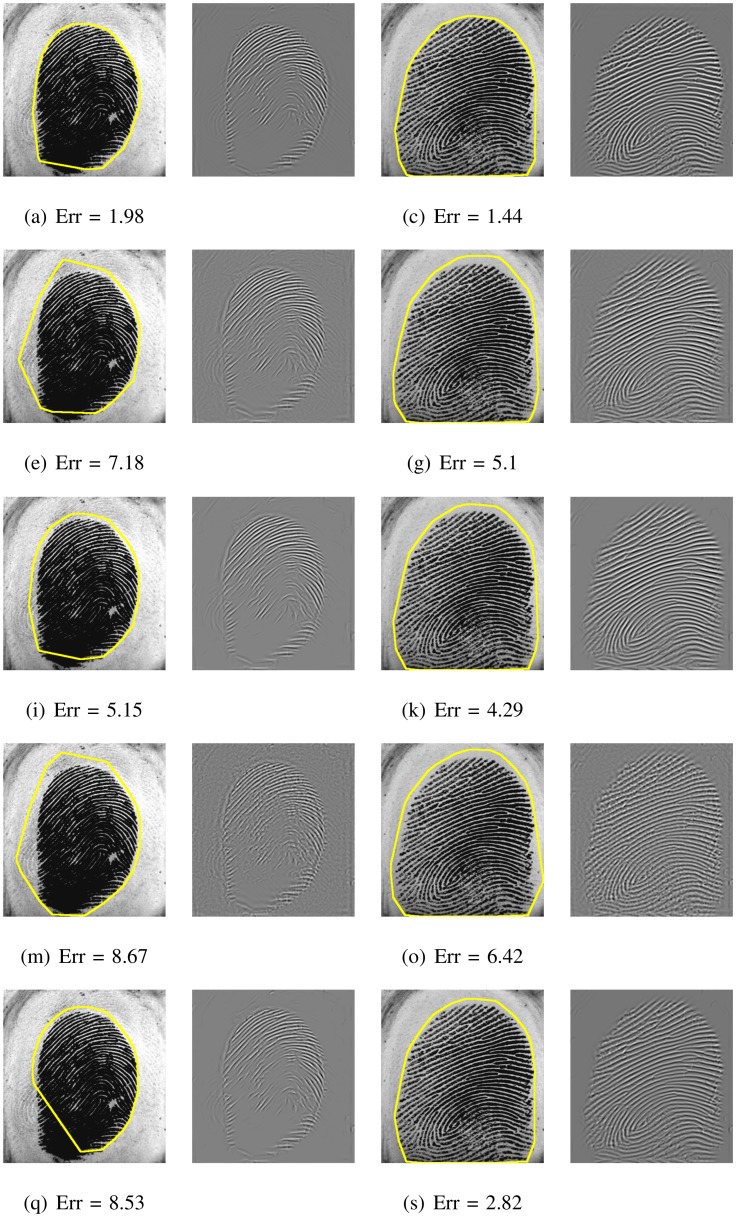
Visualization of the coefficients in the 16 subbands of the DHBB filter for*n* = 20, *γ* = 3, *ω*_*L*_ = 0.3, *ω*_*H*_ = 1.

For the proposed FDB method, the involved parameters are summarized in Table 2 and the values of the learned parameters are reported in Table 3. Also, the mirror boundary condition with size 15 pixels is used in order to avoid boundary effects. In a reasonable amount of time, a number of conceivable parameter combinations were evaluated on the training set. The choice of these parameters balances the smoothing properties of the proposed filter attempting to avoid both under-smoothing and over-smoothing. The response of a fingerprint image by FDB is illustrated in Fig 10.

**Table 2:** Overview over all parameters for the factorized directional bandpass (FDB) method for fingerprint segmentation.

Parameters	Description
*C*	a constant for selecting the threshold *β* in Eq (8) which removes small coefficients corresponding to noise.
*n*	the order of the directional Hilbert transform which corresponds to the angularpass filter in Eq (4).
*L*	the number of orientations in the angularpass filter in Eq (4).
*γ*	the order of the Butterworth bandpass filter in Eq (2).
*s*	the window size of the block in the postprocessing step in Eq (9).
*t*	a constant for selecting the morphology threshold *T* in Eq (9).
*b*	the number of the neighboring blocks in Eq (9).

**Table 3:** Overview over the parameters learned on the training set. The other four parameters are *n* = 20, *L* = 16, *s* = 9 and *b* = 6 for all databases.

FVC	DB	*C*	*γ*	*t*
2000	1	0.06	4	5
	2	0.07	2	5
	3	0.06	4	4
	4	0.03	1	5
2002	1	0.04	1	4
	2	0.05	1	7
	3	0.09	1	5
	4	0.03	1	6
2004	1	0.04	1	7
	2	0.08	2	5
	3	0.07	1	6
	4	0.05	1	5

**Fig 11:**
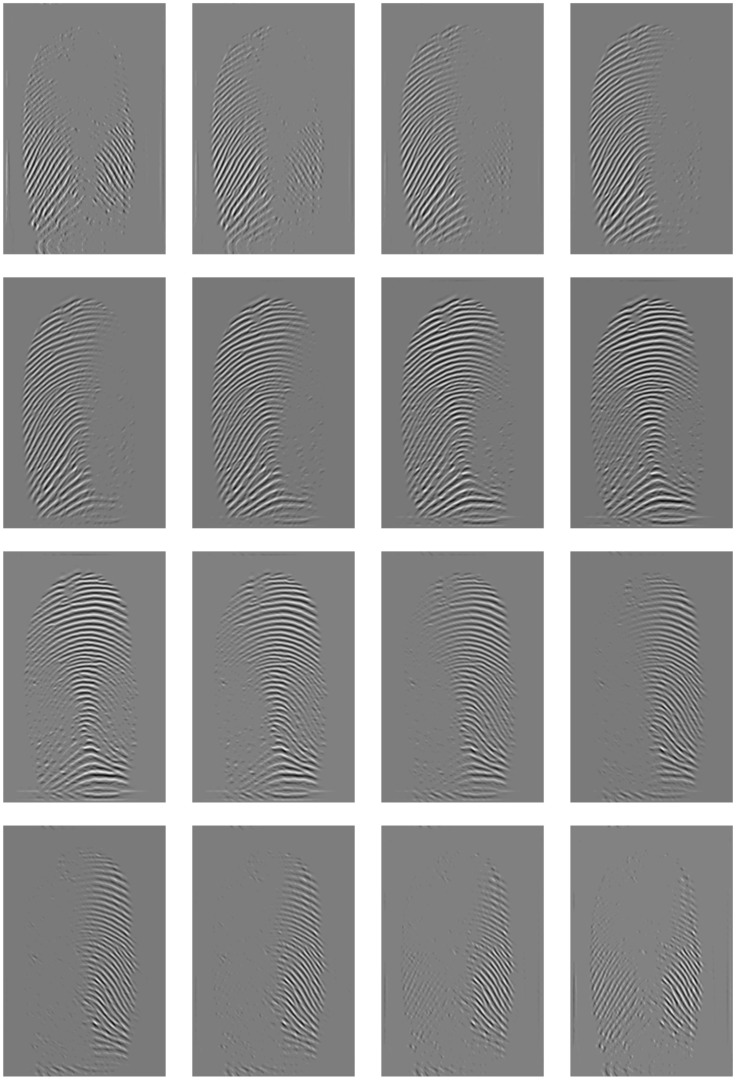
Segmented fingerprint images and the corresponding reconstructed texture images by the FDB method for FVC2000 (first and second row), FVC2002 (third and fourth row) and FVC2004 (fifth and sixth row). Columns f.l.t.r correspond to DB1 to DB4.

**Fig 12:**
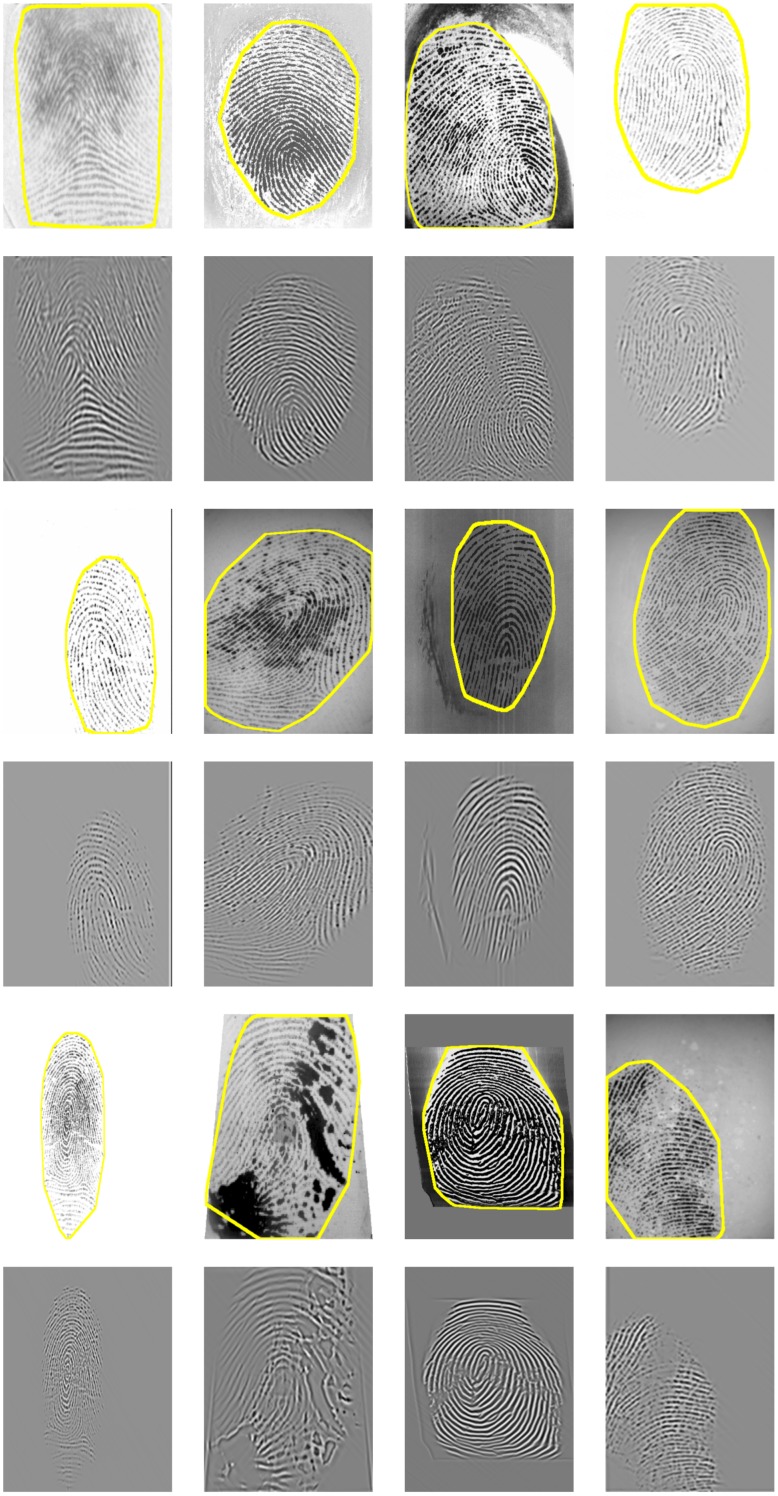
Examples of incorrectly segmented fingerprint images due to: (a) a ghost fingerprint on the sensor surface, (b) dryness of the finger, (c) texture artifacts in the reconstructed image, (d) wetness of the finger. The first row shows the segmentation obtained by the FDB method, the second row displays the reconstructed image and the last row visualizes the manually marked ground truth segmentation.

**Fig 13:**
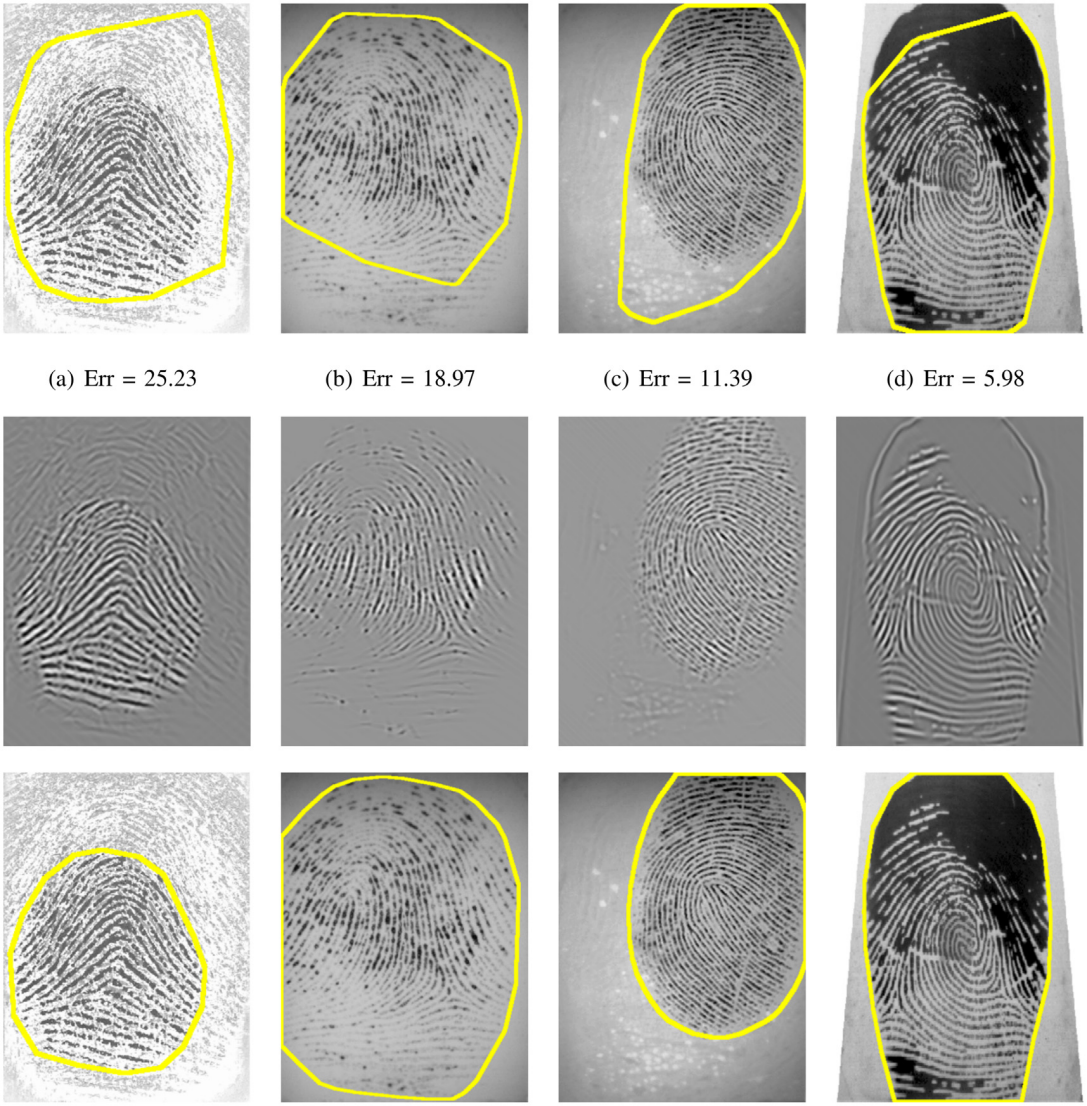
Segmented fingerprint images and their features of different methods for FVC2002_DB3_IM_15_1. (a) ground truth; (b, g) FDB, (c, h) Gabor, (d, i) Harris, (e, j, k, l) Mean-Variance-Coherence, (f, m) STFT.

**Fig 14:**
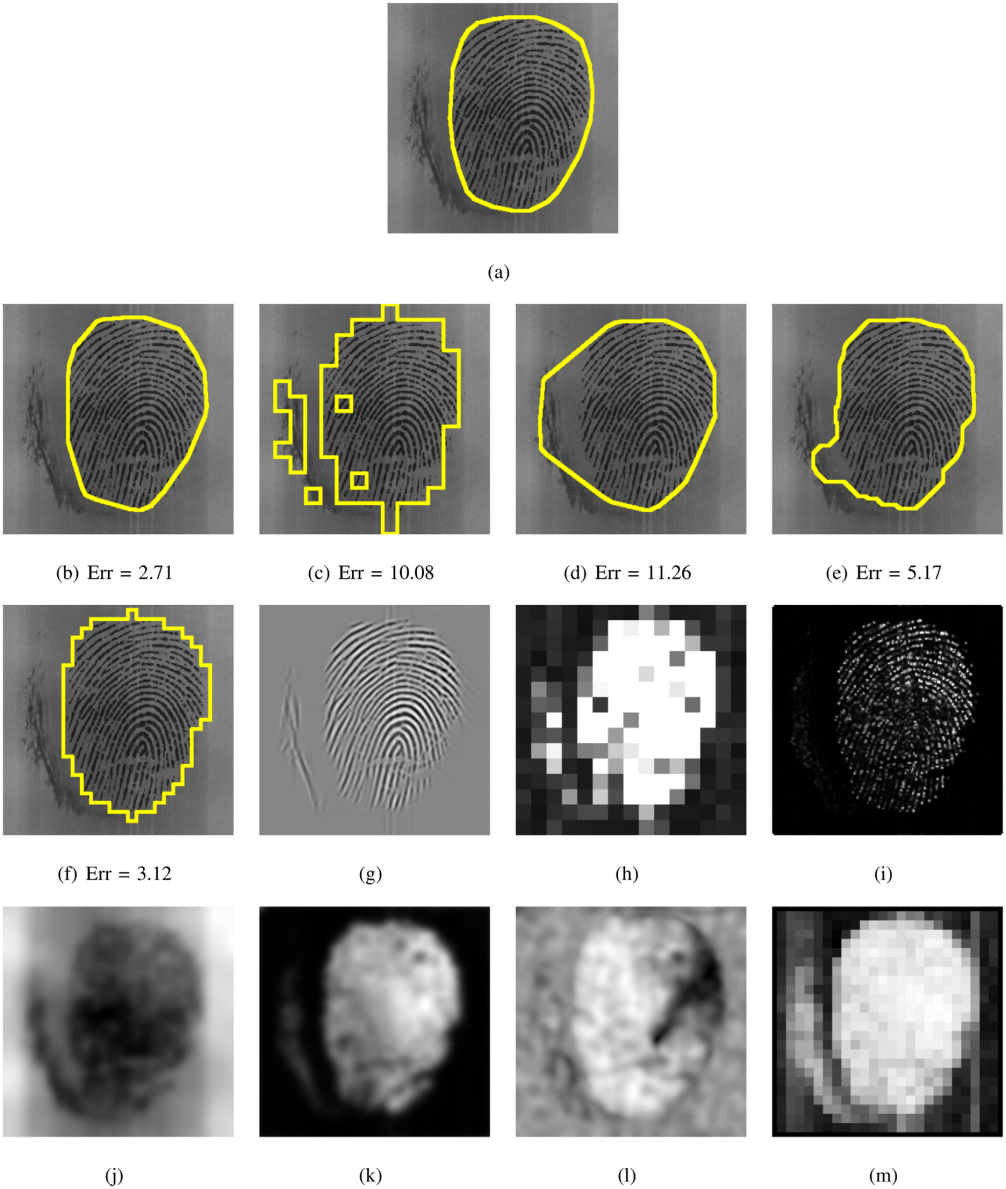
Segmented fingerprint images and their features of different methods for FVC2004_DB1_IM_24_7. (a) ground truth; (b, g) FDB, (c, h) Gabor, (d, i) Harris, (e, j, k, l) Mean-Variance-Coherence, (f, m) STFT.

**Fig 15:**
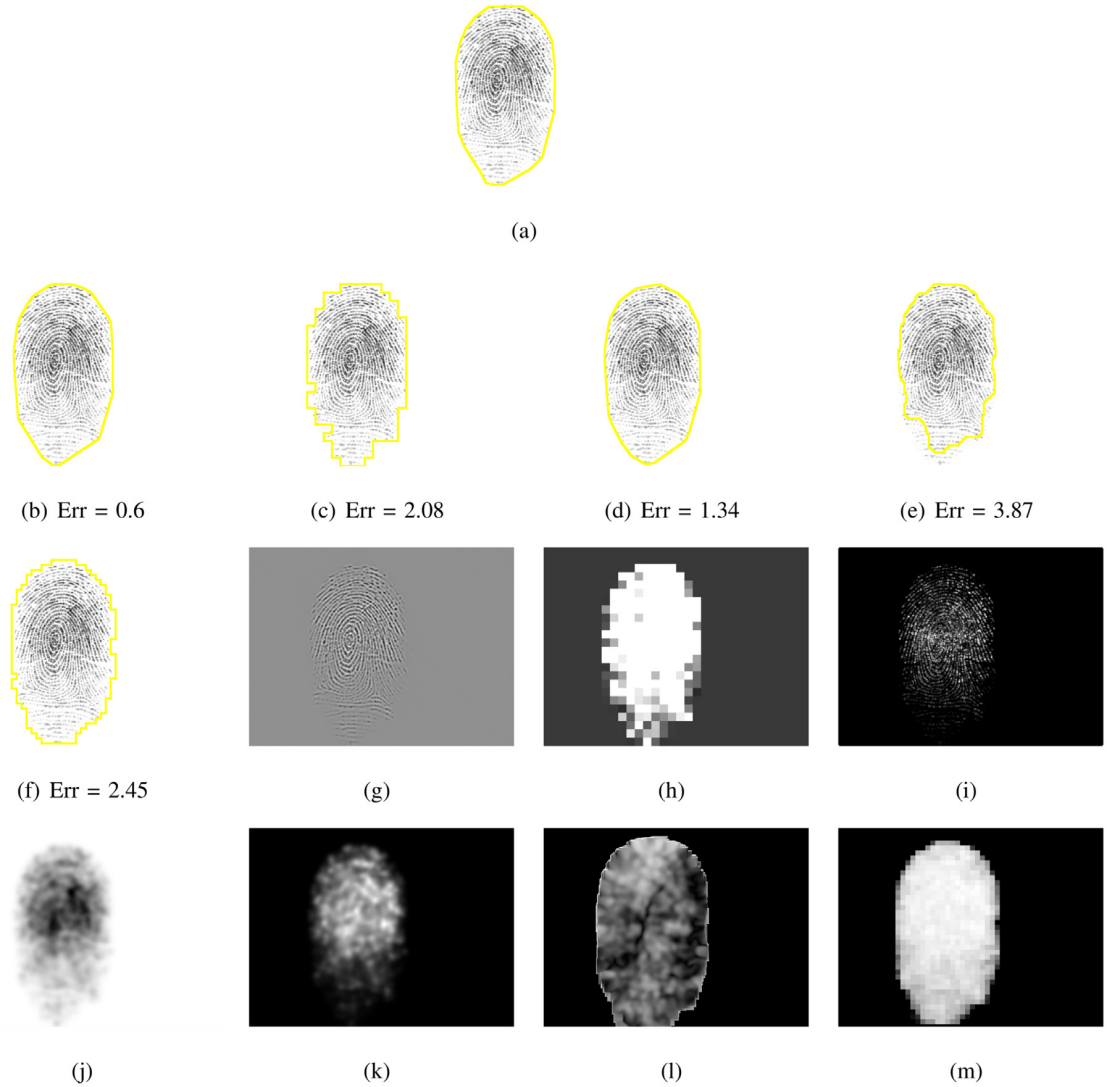
Segmented fingerprint images and their features of different methods for FVC2000_DB3_IM_17_3. (a) ground truth; (b, g) FDB, (c, h) Gabor, (d, i) Harris, (e, j, k, l) Mean-Variance-Coherence, (f, m) STFT.

**Fig 16:**
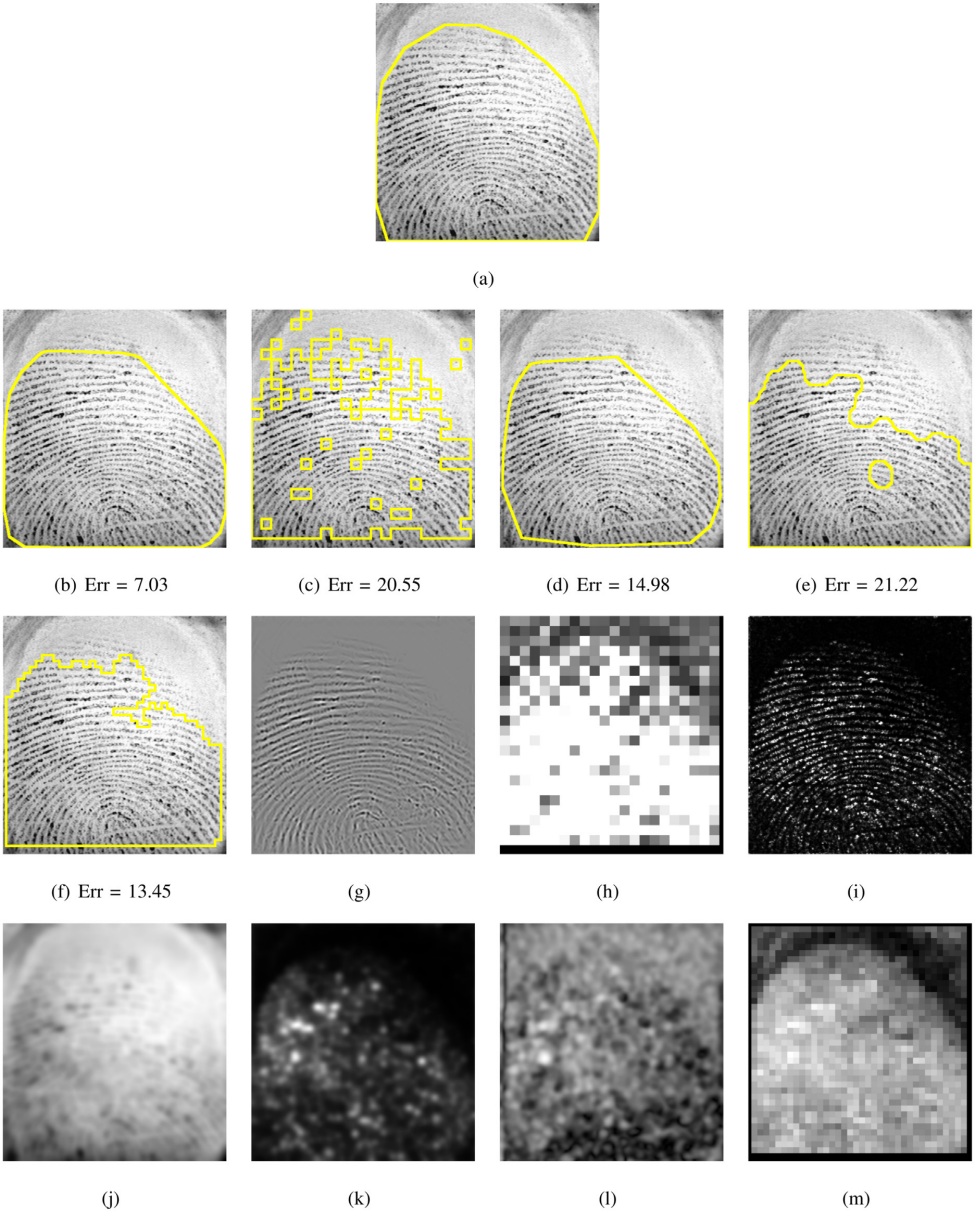
Segmented fingerprint images and their features of different methods for FVC2004_DB2_IM_56_8. (a) ground truth; (b, g) FDB, (c, h) Gabor, (d, i) Harris, (e, j, k, l) Mean-Variance-Coherence, (f, m) STFT.

**Fig 17:**
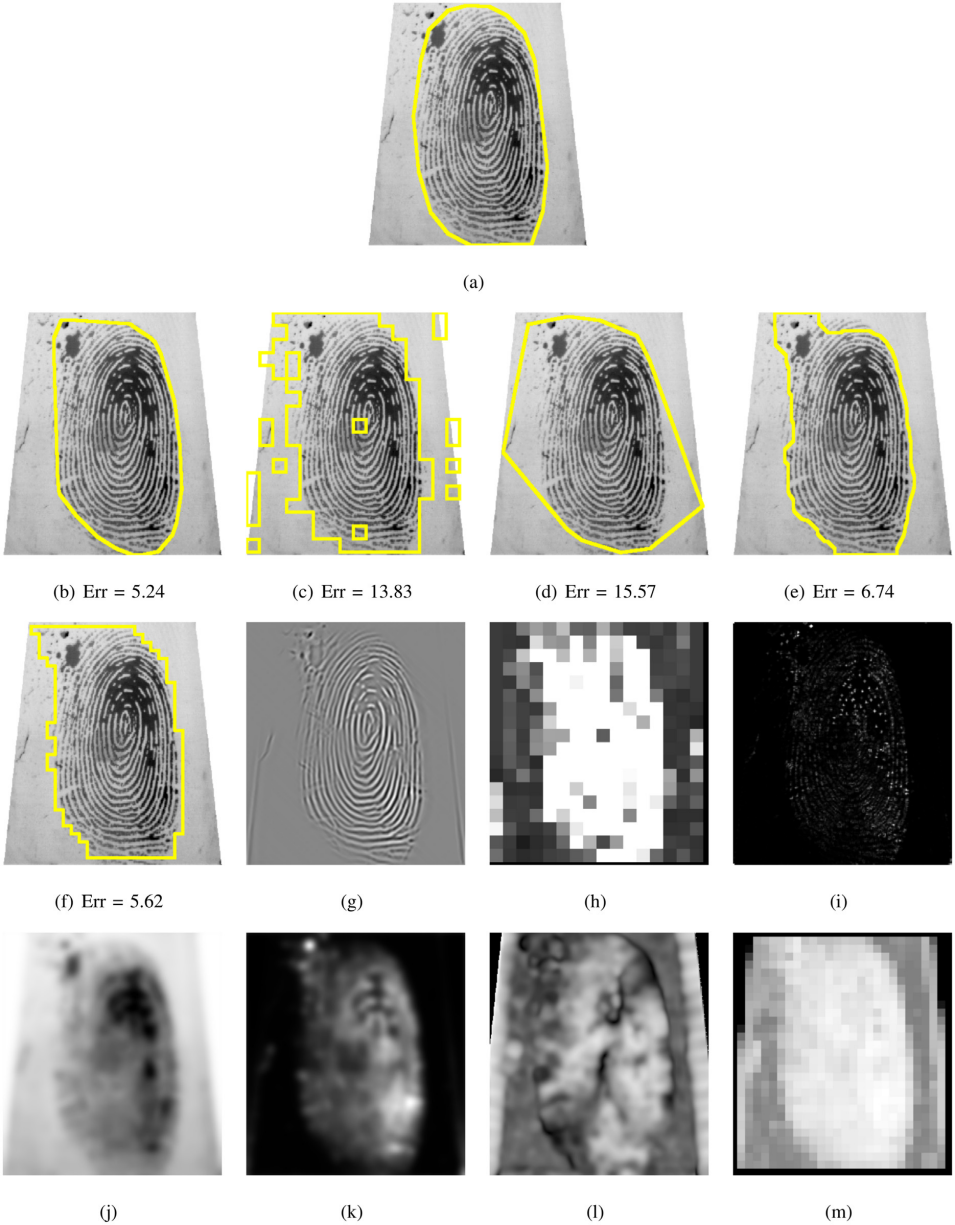
Segmented fingerprint images and their features of different methods for FVC2004_DB2_IM_65_7. (a) ground truth; (b, g) FDB, (c, h) Gabor, (d, i) Harris, (e, j, k, l) Mean-Variance-Coherence, (f, m) STFT.

This systematic comparison of fingerprint segmentation methods clearly shows that the factorized directional bandpass method (FDB) outperforms the other four widely used segmentation methods on all 12 databases. An overview of visualized segmentation results by the FDB method is given in Fig 11. A few challenging examples for which the FDB method produces a flawed segmentation are depicted in Fig 12. Moreover, a comparison of all five segmentation methods and their main features for five example images are shown in Figs 13 to 17.

## Conclusions

In this paper, we designed a filter specifically for fingerprints which is based on the directional Hilbert transform of Butterworth bandpass filters. A systematic comparison with four widely used fingerprint segmentation showed that the proposed FDB method outperforms these methods on all 12 FVC databases using manually marked ground truth segmentation for the performance evaluation. The proposed FDB method for fingerprint segmentation can be combined with all methods for orientation field estimation like e.g. the line sensor method [56] or by a global model based on quadratic differentials [57] followed by liveness detection [58] or fingerprint image enhancement [2, 59]. It can also be used in combination with alternative approaches, e.g. as a preprocessing step for locally adaptive fingerprint enhancement in the Fourier domain as proposed by Bartůněk *et al.* [20] or before applying structure tensor derived symmetry features for enhancement and minutiae extraction proposed by Fronthaler *et al.* [60].

Notably, the filter $\phi_{l}^{n,\gamma }*\phi_{l}^{n,\gamma ,\vee }$ is similar to the Gabor filter which could have been used instead of the DHBB filter. Similarly, Bessel or Chebbychev transforms as well as B-splines as generalizations ([61]) could replace the Butterworth. We expect, however, for reasons elaborated, relying on the DHBB filter gives superior segmentation results.

The manually marked ground truth benchmark is available for download at http://dx.doi.org/10.6084/m9.figshare.1294209 and the Matlab implementation of the FDB method is available for download at http://dx.doi.org/10.6084/m9.figshare.1294210.

In doing so, we would like to facilitate the reproducibility of the presented results and promote the comparability of fingerprint segmentation methods. Recently, this implementation of the FDB method has been applied to improve the performance of fingerprint liveness detection by the convolution comparison patterns [62] and fingerprint alteration detection [63]. The manually marked benchmark has been used by Thai and Gottschlich [64] and by Bartůněk [65] for evaluating a new fingerprint segmentation methods. The G3PD method [64] follows a variational approach to decompose a fingerprint image into three parts and obtains the ROI based on the texture component. The further advanced and more general DG3PD method [66] has also been applied to latent fingerprint segmentation. The method by Bartůněk [65] relies on normalisation and local kurtosis estimation as a novel feature for segmentation.

## Supplementary Appendix

### Comparison of the Operator in the FDB Method with the Summation and Maximum Operators

**Fig 18:**
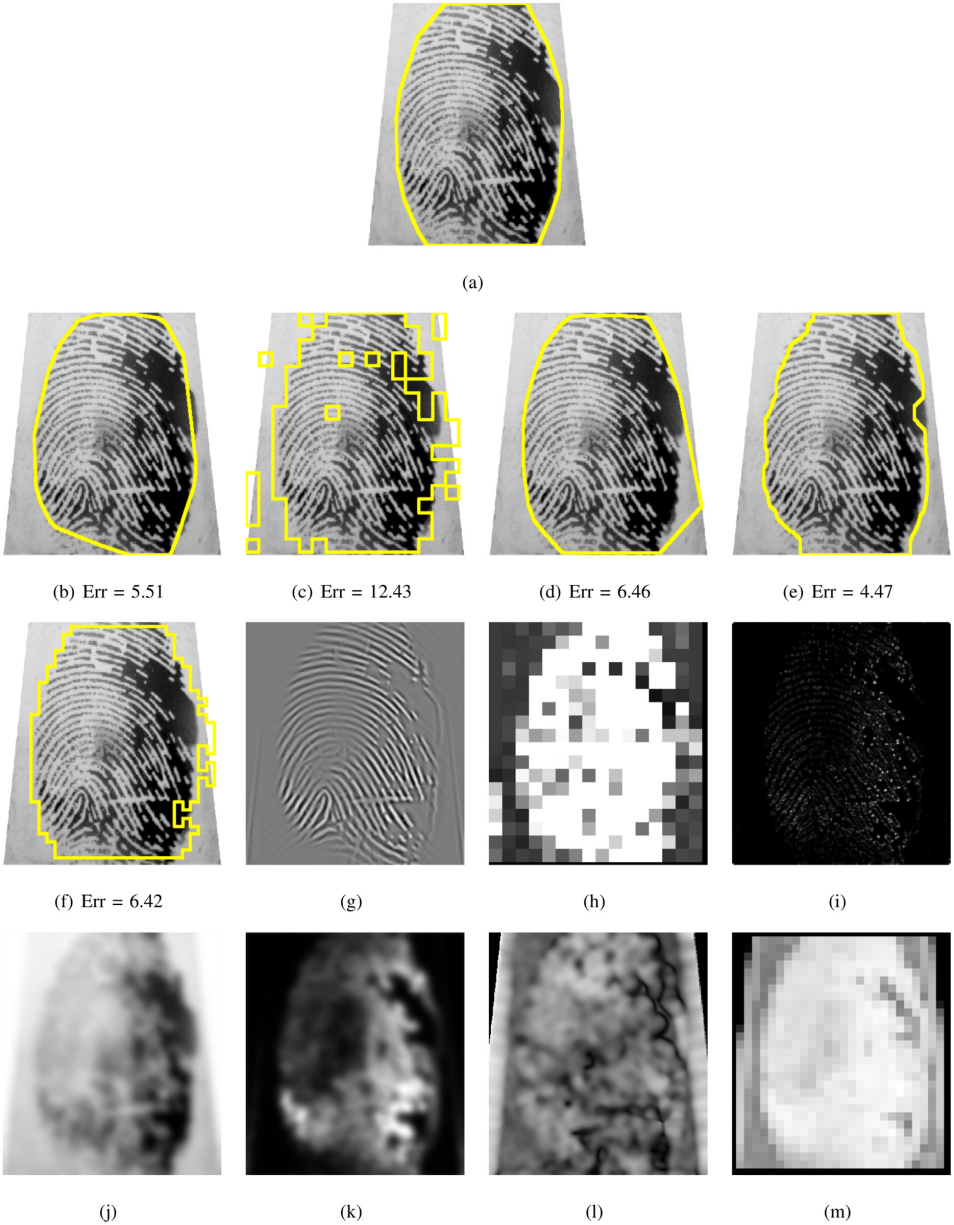
Comparison of five image reconstruction strategies and their effect on the resulting segmentation. 1^st^, 2^nd^ columns: segmented images (error in percent) and reconstructed images for a low-quality image and 3^rd^, 4^th^ columns for a good quality image. 1^st^ row: the proposed operator. 2^nd^, 3^rd^ rows: maximum operator without and with the shrinkage operator Eq (7), respectively. 4^th^, 5^th^ rows: summation operator without and with the shrinkage operator Eq (7), respectively.

We briefly illustrate the differences between the proposed FDB filter Eq (1) and the maximum and summation operators for the coefficients in all directional subbands. Fig 18 compares the results of these operators for a low-quality and a good quality example. The functions are described as follows

- The maximum operator without and with the shrinkage operator Eq (7) (depicted in the second and third row in Fig 18)
  $$\tilde{f}\left[k\right]=\left\{\begin{array}{c} \max_{l}\left\{c_{l}\left[k\right]\cdot \left(c_{l}\left[k\right]>0\right)\right\}+\min_{l}\left\{c_{l}\left[k\right]\cdot \left(c_{l}\left[k\right]<0\right)\right\}\;\left(\text{without}\;(7)\right)\\ \max_{l}\left\{d_{l}\left[k\right]\cdot \left(d_{l}\left[k\right]>0\right)\right\}+\min_{l}\left\{d_{l}\left[k\right]\cdot \left(d_{l}\left[k\right]<0\right)\right\}\;\left(\text{with}\;(7)\right), \end{array}\right.$$
  with *l* = 0, 1, …, *L* − 1.
- The summation operator without and with the shrinkage operator Eq (7) (displayed in the fourth and fifth row in Fig 18)
  $$\tilde{f}\left[k\right]=\left\{\begin{array}{c} \sum_{l=0}^{L-1}c_{l}\left[k\right]\;\left(\text{without}\;(7)\right)\\ \sum_{l=0}^{L-1}d_{l}\left[k\right]\;\left(\text{with}\;(7)\right). \end{array}\right.$$
